# Mechanisms of Drug Resistance in Ovarian Cancer and Associated Gene Targets

**DOI:** 10.3390/cancers14246246

**Published:** 2022-12-18

**Authors:** Kharimat Lora Alatise, Samantha Gardner, Angela Alexander-Bryant

**Affiliations:** Nanobiotechnology Lab, Department of Bioengineering, Clemson University, Clemson, SC 29634, USA

**Keywords:** chemoresistance, ovarian cancer, gene targets, RNAi therapeutics, drug resistance

## Abstract

**Simple Summary:**

When tumors become resistant to chemotherapeutics, alternative treatment strategies must be explored. Gene targeting provides a personalized and molecular approach to tackling chemoresistance in ovarian cancer. However, to advance the current landscape of gene targeting in ovarian cancer, the therapeutic potential of more gene targets should be explored. Here, we review several novel and well-studied genes that can be investigated as potential gene targets in ovarian cancer to increase chemotherapeutic response.

**Abstract:**

In the United States, over 100,000 women are diagnosed with a gynecologic malignancy every year, with ovarian cancer being the most lethal. One of the hallmark characteristics of ovarian cancer is the development of resistance to chemotherapeutics. While the exact mechanisms of chemoresistance are poorly understood, it is known that changes at the cellular and molecular level make chemoresistance challenging to treat. Improved therapeutic options are needed to target these changes at the molecular level. Using a precision medicine approach, such as gene therapy, genes can be specifically exploited to resensitize tumors to therapeutics. This review highlights traditional and novel gene targets that can be used to develop new and improved targeted therapies, from drug efflux proteins to ovarian cancer stem cells. The review also addresses the clinical relevance and landscape of the discussed gene targets.

## 1. Introduction

Ovarian cancer is the most lethal gynecological malignancy [1,2]. In 2022 alone, it is estimated there will be 19,880 new cases and 12,810 deaths due to ovarian cancer in the United States [3]. The survival rate of patients with ovarian cancer is related to the disease stage at diagnosis. Women diagnosed with localized disease (stage 1) have an average 5-year survival rate of 92.6% [4], whereas women diagnosed in the later metastatic stages (Stage 3 and 4) have an average 5-year survival rate of only 30.2% [4]. Approximately 70% of all ovarian cancer diagnoses occur in advanced stages, reducing the patients’ overall survival rate [5]. The presentation of nonspecific symptoms combined with limited detection and screening methods contributes to the high percentage of women diagnosed in advanced stages [2].

The standard treatment method for ovarian cancer is debulking surgery followed by chemotherapy and/or radiation therapy. Another common treatment method is neoadjuvant chemotherapy followed by debulking surgery [1]. Platinum- and taxane-based drugs are typically used to treat ovarian cancer; however, the selection of chemotherapeutic agents depends on the stage of ovarian cancer [1]. High-dosage chemotherapy often leads to complications as well as chemotherapeutic resistance; over 70% of patients relapse after treatment and eventually become resistant to chemotherapeutics [6]. Generally, chemotherapeutic resistance is a phenomenon that occurs when a disease becomes tolerant to a therapeutic over time, thus reducing the efficacy of the drug. Resistance in cancers can be characterized as either intrinsic or acquired resistance. Intrinsic resistance indicates that there are pre-existing factors within tumor cells that make initial chemotherapeutic treatments less effective. Mechanisms of intrinsic resistance involve drug degradation by drug-metabolizing enzymes, mutations in the drug target, and modifications in membrane transport of the drug. Poor vascularization, extracellular matrix (ECM) interactions, and cellular metabolic processes are also contributing factors to intrinsic resistance [7,8]. Acquired resistance, however, is developed after treatment with therapeutics, implying an increase in mutations and alterations within the tumor cells in response to chemotherapy which limits drug efficacy. Acquired resistance can be attributed to increased drug efflux due to the overexpression of drug efflux proteins, activation of survival signaling pathways, and inactivation of DNA damage repair mechanisms to evade cell death [9,10]. Both intrinsic and acquired resistance are multifactorial and often involve various independent and dependent pathways, making treatment complex [9]. Nevertheless, tackling resistance is a critical clinical need to improve outcomes for patients with ovarian cancer.

Although the exact molecular mechanisms behind resistance in ovarian cancer are poorly understood, it is recognized that patients with ovarian cancer develop acquired resistance from platinum- and taxane-based therapeutics. Analyzing the differences in protein expression in chemosensitive and chemoresistant ovarian cancer can give rise to new therapeutic targets. Protein expression levels vary in cellular processes such as apoptosis, DNA repair, and the cell cycle in resistant ovarian cancer. For example, upregulated proteins can have inhibitory actions on apoptosis, while downregulated proteins that usually trigger apoptosis may no longer function at their full capacity. In this review, we examine an array of proteins that have been linked to chemoresistance in ovarian cancer, particularly proteins associated with drug efflux, inhibition of apoptosis, DNA damage and repair, and cancer stem cells. While common genes have been targeted through singular or combinatorial therapy, targeting a wider range of genes using RNA interference (RNAi) tools may be effective in providing a more personalized medicine approach. Here, we discuss pathways and mechanisms of chemoresistance in ovarian cancer and associated gene targets that can be explored for targeting and therapeutic approaches.

## 2. Drug Efflux Proteins

**Figure 1:**
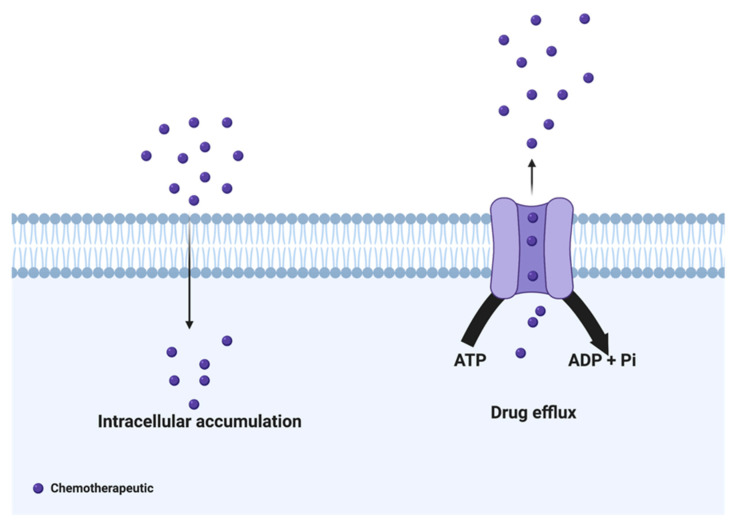
Generic scheme of drug efflux proteins. The adenosine triphosphate (ATP) Binding Cassette (ABC) family of membrane proteins enables the efflux of therapeutics. ABC transporters (light purple) use ATP to pump chemotherapeutics (dark purple) out of the cell.

Drug efflux proteins are central in the development of therapeutic resistance in ovarian cancer. Chemotherapeutics must be delivered into the cell’s cytoplasm for maximum therapeutic benefit; however, efflux proteins can remove various drugs from the cell (Figure 1). The most notable drug efflux and membrane transporter proteins linked to resistance are among the adenosine triphosphate-binding cassette (ABC) superfamily. ABC transporters use adenosine triphosphate (ATP) to move substrates across the cellular membrane [11,12]. Proteins of this family all follow a similar basic structure—they are composed of two cytoplasmic nucleotide-binding domains and two transmembrane domains responsible for binding to and hydrolyzing ATP and recognizing transport molecules, respectively [13,14]. Within this large family of proteins, seven subfamilies have been categorized from A to G based on sequence homology [15]. Specifically, *ABCB1* (P-glycoprotein/PgP, multidrug resistance protein 1/MDR1), *ABCC1* (multidrug resistance-associated protein 1/MRP1), and *ABCG2* (Breast Cancer Resistance Protein/BCRP) have been linked to chemoresistance [15].

While there are over 40 proteins associated with the ABC transporter family, *ABCB1* is one of the most commonly studied [7,10,16]. *ABCB1* is a 170 kDa unidirectional membrane-bound glycoprotein known to reduce the concentration of platinum- and taxane-based chemotherapeutics in ovarian cancer cells [17,18]. Studies have shown that *ABCB1* expression is a prognostic factor in ovarian cancer [19]. The protein is also overexpressed in chemoresistant ovarian cancer cells, specifically in cells treated with paclitaxel and cisplatin [20,21]. Overcoming resistance mediated by *ABCB1* has been studied over the past three decades with an emphasis on delivering small inhibitor molecules and small interfering RNAs to reduce its expression [17]. Previous studies have shown that knockdown of the *ABCB1* gene can re-sensitize and increase the intracellular accumulation of chemotherapeutics in drug-resistant ovarian cancer cells, making it a suitable target for treatment [22,23].

*ABCC1* is a 190 kDa glycophosphoprotein that is not only active in drug transport but also in the transportation of conjugated organic anions such as glutathione and glucuronate [24,25,26]. The protein was discovered in a multidrug-resistant lung cancer cell line that did not overexpress *ABCB1* [27]. *ABCC1* is thought to induce an inflammatory response as well as protect cells from oxidative stress, xenobiotics, and endogenous toxic metabolites. However, high expression of *ABCC1* also plays a role in disease progression and drug resistance in ovarian cancer [25,28,29]. In a study conducted by Ohishi et al., it was revealed that *ABCC1* mRNA was elevated in untreated and cisplatin/carboplatin-treated ovarian carcinoma tumor samples from patients with progressive ovarian cancer [30]. Due to the elevated expression of *ABCC1* in both untreated and treated samples, these results suggest that *ABCC1* could be involved in intrinsic and acquired resistance. Similarly, increased expression of *ABCC1* transcripts was found in ovarian cancer tissue before chemotherapy treatment compared to normal (healthy) ovarian tissue [31]. Downregulation of the *ABCC1* gene in vitro has resulted in increased sensitivity to various chemotherapeutics and decreased cell proliferation in cancers such as glioblastoma multiforme, and lung, colorectal and esophageal cancer [32,33,34,35]. One research group has described using an *ABCC1* small molecule inhibitor in combination with a glutathione-depleting drug to explore cellular viability and chemosensitization in SKOV3 ovarian cancer cells where combination treatment displayed a loss of viability [36]. To our knowledge, *ABCC1* has not been targeted using RNAi-based therapies specifically for the treatment of drug resistance.

*ABCG2* is considered a half-transporter with a molecular weight of 72 kDa. Although it is half the size of *ABCB1* and *ABCC1*, it has been shown to act similarly and i*s* composed of at least one nucleotide-binding domain and two transmembrane domains [37,38]. While it was initially isolated from a breast cancer cell line, increased expression of *ABCG2* has been identified in many cancers, including myeloma, glioblastoma, esophageal, tongue, and ovarian cancer [39,40]. *ABCG2* upregulation is correlated with resistance to topoisomerase inhibitors, anthracyclines, and mitoxantrone [41,42]. In ovarian cancer, overexpression of *ABCG2* has been revealed through elevated mRNA transcript levels in topotecan-resistant A2780 and IGROV1 cells [43,44]. Because most studies analyze chemoresistance in cell lines resistant to one type of chemotherapeutic, Januchowski et al. sought to study acquired drug resistance by multiple chemotherapeutics. Their studies observed cross-resistance between doxorubicin-treated and topotecan-treated A2780 cell lines, indicating that *ABCG2* overexpression is related to both doxorubicin and topotecan resistance [44]. The role of *ABCG2* was further analyzed by Mo et al., where it was determined that in ID8 murine ovarian cancer cells, the inhibition of ABCC2 and *ABCG2* via small molecule drugs MK-571 and Novobiocin decreased the efflux of Rhodamine 123, a tracer dye that can bind to ABC transporters [45]. Based on these results, it was revealed that in ascites-derived human ovarian cancer cells, *ABCC1* and *ABCG2* promote drug efflux. Lastly, Ricci et al. demonstrated that three different *ABCG2* inhibitors each restored chemosensitivity in topotecan-resistant IGROV1/T8 cells in vitro and in vivo with minimal cytotoxic effects [46]. There are limited studies targeting the *ABCG2* gene for ovarian cancer, even though it is being studied clinically in other cancers; however, there is a correlation between *ABCG2* upregulation and resistance to anthracyclines, such as doxorubicin. Targeting *ABCG2* could be beneficial for patients who have experienced relapse and resistance to other drugs, especially since anthracyclines are typically used as a second line of therapy for patients resistant to other chemotherapeutics.

Though targeting efflux proteins biases cells to take in more of a drug, reducing the expression of efflux proteins alone is insufficient. Increasing drug uptake does not always result in maximum therapeutic benefit and outcomes, especially with multifactorial drug resistance. For optimal results in overcoming drug resistance, it is best to target drug efflux proteins in combination with another gene/pathway involved in resistance to increase the efficacy of the drug.

## 3. Apoptosis

**Figure 2:**
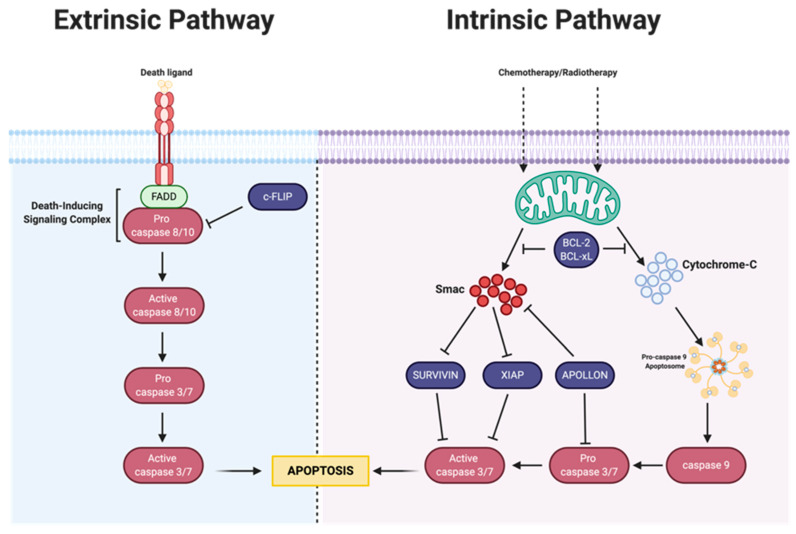
The intrinsic and extrinsic apoptosis pathways. The extrinsic apoptosis pathway is initiated through ligand–receptor interactions. The intrinsic pathway is mediated by the release of *Smac* and cytochrome C from mitochondria (green). Inhibitory proteins (dark purple) can interrupt the caspase cascade (pink), ultimately preventing apoptosis.

Most anticancer agents are meant to trigger cell death through various mechanisms, such as apoptosis. However, resistance can develop when apoptosis is delayed or inhibited, which reduces the efficacy of the drug. Because chemotherapy is known to induce apoptosis within the cell, deficiencies within the apoptotic pathway can lead to resistance [47]. The suppression of apoptosis is linked with the progression of ovarian cancer as well as other cancers [1]. Apoptosis is initiated through both intrinsic and extrinsic pathways. Intrinsic apoptosis is mitochondrial-dependent and mediated by stress signals at the mitochondrial level. These stress signals can cause intracellular damage due toradiation, hypoxia, oxidative stress, and/or treatment with chemotherapeutics, triggering the release of cytochrome c, an apoptosis signaling protein, from mitochondria [48,49]. In contrast, the extrinsic apoptotic pathway is mediated by extracellular signals and receptors that belong to the tumor necrosis factor superfamily [48,49]. Though independent of one another, both pathways utilize caspases to initiate, execute and regulate the apoptosis cascade (Figure 2) [50]. In drug-resistant ovarian cancer, the expression of anti-apoptotic proteins is exacerbated post-treatment; these anti-apoptotic proteins can hinder the initiation of apoptosis by indirectly or directly blocking the caspase cascade.

Generally, apoptosis is mediated by a family of cysteine–aspartyl proteases (caspases). Caspases are inactive enzymes comprised of subunits and become activated once their peptide bonds are hydrolyzed, eventually separating the subunits from one another [51]. Interaction with one caspase triggers the activation of another, which is often referred to as the caspase cascade. In chemoresistant ovarian cancer, modulation of the caspase cascade can lead to inhibition of cell death, resulting in the progression of the disease. A clinical study demonstrated that ovarian cancer patients who had tumors with low levels of expression of caspase-8 had reduced survival rates, whereas patients with higher levels of caspase-8 had longer survival rates [52]. Because caspase-8 is an initiator caspase, reduced expression affects the overall caspase cascade, which promotes cellular survival instead of cell death. In the following subsections, we discuss various proteins that affect the caspase cascade.

### 3.1. Intrinsic Apoptosis

#### 3.1.1. Bcl-2 Family

B cell lymphoma gene 2 (*Bcl-2*), one of many families in the intrinsic pathway, comprises over 20 pro- and anti-apoptotic proteins that prevent the release of cytochrome C from mitochondria [53]. Overexpression of Bcl-2 proteins can counteract the function of pro-apoptotic proteins and promote cell survival [54]. In ovarian cancer, the anti-apoptotic proteins *Bcl-2* and *Bcl-2*-related gene long isoform (*Bcl-xL*) are often upregulated and correlated with poor prognosis of the disease [55,56,57,58]. Analyzing ovarian cancer tissue samples of patients treated with cisplatin-based chemotherapy, Mano et al. found that *Bcl-2* expression was associated with a poor response, thus identifying the gene as an important prognostic factor [58]. *Bcl-2* negative samples responded to chemotherapy, thus exemplifying the activity of *Bcl-2* in promoting ovarian cancer cell survival. In another study, Yang et al. developed chemoresistant SKOV3 and OVCAR3 ovarian cancer spheroids to determine the underlying mechanism behind platinum resistance within the spheroids [59]. The spheroids exhibited increased expression of *Bcl-2* compared to ovarian cancer cells cultured in monolayers [59]. Downregulation of *Bcl-2* using small interfering RNA (siRNA) enhanced cell death in the spheroids, demonstrating resensitization to cisplatin [59]. This work confirms the importance of *Bcl-2* in ovarian cancer and validates its role as a key gene in mediating drug resistance via apoptotic suppression.

Similar evidence relating the structural and functional homolog of *Bcl-2*, *Bcl-xL*, to drug resistance in ovarian cancer was found in a study by Brotin et al. [55]. The study revealed that *Bcl-xL* protected cisplatin-resistant SKOV3 cells from apoptosis, and silencing the *Bcl-xL* gene using siRNA with cisplatin treatment induced apoptosis [55]. Degradation of DNA in ovarian carcinoma can be a sign of apoptotic cell death, usually caused by chemotherapeutics treatment. The overexpression of *Bcl-xL* can delay and prevent the activation of apoptosis in ovarian cancer, allowing more time for DNA repair. Liu and colleagues suggested that a delayed response in apoptosis may allow cells to develop another mechanism of resistance in addition to increased DNA repair, but it is unknown whether knockdown of *Bcl-xL* causes pleiotropic drug resistance [60]. If *Bcl-xL* does cause pleiotropic drug resistance, further studies should be done to determine what genes are upregulated or downregulated after *Bcl-xL* knockdown.

While proteins in the Bcl family have been targeted in ovarian cancer, plenty is still unknown. Elucidating the downstream effects of targeting Bcl family proteins, aside from triggering apoptosis, will help uncover more about the role the Bcl family plays in drug resistance and cell survival mechanisms. In addition, RNAi-based therapeutics for targeting the Bcl family in ovarian cancer are limited. Primarily small molecule drugs are being used to target Bcl in current therapeutic strategies.

#### 3.1.2. *IAP* Family

While the *Bcl-2* superfamily plays a significant role in the intrinsic apoptosis pathway, the inhibitor of apoptosis protein (IAP) family also contributes to apoptosis inhibition. IAPs are anti-apoptotic proteins and ubiquitin ligases that bind to caspases, resulting in inhibition or degradation [61]. IAPs are only functional when they are not bound to second mitochondria-derived activator of caspase (*Smac*), a protein that inhibits their mechanism of action [62]. *Smac* is a mitochondrial intermembrane space protein that can induce apoptosis [50,63]. *Smac* has been shown to activate caspase-9, and similar to cytochrome C, it is released from mitochondria into the cytosol, causing a downstream signaling cascade to initiate apoptosis [62,64]. In drug-resistant ovarian cancer, IAPs are expressed at higher levels than *Smac*, inhibiting the apoptosis-inducing activity of *Smac* and ultimately leading to chemoresistance [49,50]. Targeting this family of proteins could also play an essential role in inflammation, cell survival, and regulating major cell signaling pathways in ovarian cancer [65].

The IAP family is comprised of eight proteins, which include the X-linked inhibitor of apoptosis protein (XIAP), Survivin, and Apollon [66]. XIAP can prevent and regulate apoptotic cell death by directly binding to and inhibiting caspase-3, -7, and -9, the last caspases in the signaling cascade that leads to apoptosis [67]. As a result, the expression of *XIAP* has been linked to chemoresistance in established ovarian cancer cell lines and primary cultures [68,69,70,71]. In fact, work done by Sapi et al. demonstrated that resistance to docetaxel in SKOV3 and primary ovarian cancer cells was mediated by increased expression of *XIAP* [68]. Modulating *XIAP* expression using RNAi enabled caspase 3 activation and the apoptosis of ovarian cancer cells treated with docetaxel [68]. Similar results obtained by Ma et al. demonstrated that downregulation of the *XIAP* gene in ovarian cancer resulted in chemosensitization, which reduced A2780/cp70 cell proliferation in vitro and tumorigenicity in vivo in BALB/c nude mice through the induction of apoptosis [72]. Unlike other IAPs, *XIAP* has a direct affinity to caspases, making it a noteworthy target. Using gene therapies to target *XIAP* would completely degrade the protein rather than using a small molecule antagonist that may or may not have selective binding [73]. Because of the multiple domains in the structure of *XIAP*, small-molecule drugs may only bind to a singular domain, reducing the efficacy of the protein. Currently, researchers are designing molecules that have specific binding to *XIAP*’s multiple domains [73]. Using gene therapies such as antisense oligonucleotides, siRNA, and shRNA would reduce the overall expression of the protein by diminishing mRNA levels.

Survivin, another protein in the IAP family, is expressed in lung, endometrial, breast, colorectal, and ovarian cancers [74,75,76]. Survivin is a 16.5 kDa protein comprised of 142 amino acids, making it the smallest member of the IAP family [75]. As a multifunctional protein, Survivin has been associated with cytoprotection and regulation of cell division [77,78,79]. The depletion of Survivin causes defects in cell proliferation and apoptosis [77]. Survivin has been found to regulate spindle checkpoints, localize mitotic spindle microtubules, and is known to have centrosomal functions and kinetochore localization [80,81,82,83,84]. For a chemotherapeutic such as paclitaxel, a microtubule-stabilizing agent, Survivin could directly impact its functionality. Increased Survivin expression inhibited taxol-induced apoptosis in ovarian cancer tumor tissue, demonstrating an inverse relationship between Survivin and taxol sensitivity [85]. To increase paclitaxel sensitivity, Kar et al. explored the treatment of ovarian cancer cells derived from ascitic fluid of primary untreated tumor samples with anti-Survivin siRNA and revealed that post Survivin knockdown, cell survival decreased by over 20% [86]. However, the exact mechanism by which Survivin inhibits apoptosis is still being explored. Several studies have investigated Survivin’s role in suppressing caspase activity, but many of these studies have shown contradictory results [84,87,88,89,90]. It has been reported that Survivin directly binds to and suppresses caspases-3, -7, and -9; however, other studies have described direct interaction between Survivin and Smac to inhibit apoptosis [87,91]. Regardless, Survivin is believed to be a regulator of mitochondrial-dependent apoptosis [92]. Additionally, there is a clear correlation between the expression of Survivin and poor prognosis in cancer, and in ovarian cancer, Survivin expression can serve as a useful prognostic and predictive marker. Specifically, in malignant ovarian carcinomas, expression levels of Survivin have been detected in 51.1–92% of patient samples and less than 25% of benign samples [93,94,95,96]. Because Survivin can be detected in most malignant samples and has an association with paclitaxel resistance, there is strong therapeutic potential for targeting Survivin to increase apoptosis in ovarian cells.

Apollon, also known as baculoviral IAP repeat-containing 6 (*BIRC6*) or BIR-containing ubiquitin-conjugating enzyme (*BRUCE*), is a 530 kDa protein, the largest among the IAP family [97]. Apollon was initially found to be upregulated in brain gliomas resistant to DNA damaging agents and antisense oligonucleotides [97]. Although the physiological role of Apollon in apoptosis remains vague, it was revealed that Apollon has ubiquitin-conjugating activity and facilitates the degradation of Smac and caspase-9, thus preventing *Smac*-induced apoptosis [98,99]. Qiu et al. reported complimentary evidence demonstrating that Apollon binds to procaspase-9 and inhibits its cleavage, which ultimately interferes with the downstream signaling of the caspase cascade [100]. Elevated Apollon expression has been identified in many cancers, including prostate, lung, colorectal, brain, esophageal, and ovarian cancers [97,101,102,103,104,105]. Apollon expression has also been linked to chemoresistance and poor prognosis. In ovarian cancer, Apollon protein expression is significantly higher in patient-derived ovarian carcinoma tissues in comparison to normal tissues [105]. Interestingly, patients in the study were not exposed to any anticancer treatment before resection of the tissue, suggesting the high expression of Apollon was intrinsic [105]. Evidence in breast cancer shows that Apollon knockdown may induce apoptosis and sensitize cells to chemotherapeutics, demonstrating its therapeutic potential [106,107]. The therapeutic benefit of targeting Apollon in breast cancer warrants the exploration of Apollon as a target in resistant ovarian cancer, as it is the least-studied IAP family member. Additionally, in ovarian cancer, not much research has focused on Apollon as a therapeutic target. However, based on the results of studies in other cancer models, it would be beneficial to explore the mechanisms of resistance caused by Apollon in ovarian cancer, as well as the downstream effects of its knockdown.

### 3.2. Extrinsic Pathway

The extrinsic apoptosis pathway, also known as the death receptor pathway, is mediated by interactions with cell surface receptors belonging to the tumor necrosis factor (TNF) family, which causes a downstream of events leading to apoptosis. This pathway serves as a connection between extracellular surroundings, such as the tumor microenvironment, and intracellular signaling networks [62]. These receptors include but are not limited to tumor necrosis factor receptor 1 (TNFR1), Fas ligand (Fas-L; APO-1 and CD95), and TNF-related apoptosis-inducing ligands TRAILR1 (Death receptor 4; DR4) and TRAILR2 (Death receptor 5; DR5). Generally, once ligands bind to their corresponding receptor on the cell surface, oligomerization of the receptors, recruitment of the Fas-associated death domain protein, activation of procaspase-8, and formation of the death-inducing silencing complex occurs, which in turn stimulates signaling to initiate apoptotic activity [108,109]. However, in ovarian cancer, these receptors can be downregulated and susceptible to resistance by treatment with their corresponding ligand, consequently suppressing downstream signaling in both instances [110]. These receptors can be exploited for ovarian tumor targeting; however, the intracellular proteins, MAPK-activating death domain protein and cellular FLICE-like inhibitory protein, which are involved in this pathway, are overexpressed in ovarian cancer, and may serve as useful targets within the extrinsic apoptosis pathway.

#### MAPK-Activation Death Domain Protein and cFLIP

MAPK-activating death domain protein (*MADD*) and cellular FLICE-like inhibitory protein (*c-FLIP*) disrupt downstream events that trigger apoptosis. *MADD* is an essential protein for cellular survival and inhibits the activation of caspase-8 [111,112]. Additionally, *MADD* is phosphorylated and binds to death receptors [112,113,114]. Many studies have focused on the necessity of *MADD* in apoptosis because the loss of *MADD* expression has been shown to increase cellular proliferation and metastasis in thyroid, cervical, breast, lung, and ovarian cancer [111,115,116,117,118]. In malignant ovarian tissues, *MADD* is expressed at significantly higher levels than in normal ovarian tissues [119]. To our knowledge, there have not been any studies examining the role *MADD* plays as a mediator of chemoresistance in ovarian cancer; however, knockdown of *MADD* in other cancers has been investigated. In breast cancer specifically, *MADD* knockdown stimulated doxorubicin- or TRAIL-induced apoptosis through the activation of caspase-8. [117]. Using siRNA, Saini et al. demonstrated that silencing *MADD* inhibited the proliferation of thyroid cancer cells in vitro and in vivo. The results also demonstrated the potential anti-migratory/invasive effects of silencing *MADD* due to a decrease in mitochondrial length, which may influence overall mitochondrial function [115]. While *MADD* has not been extensively studied in drug-resistant ovarian cancer, it is known to be a splice variant of the insulinoma-glucagonoma clone 20 (IG20) gene, which has been studied [113]. The IG20 gene encodes for four different splice variants, including *MADD*. Studies examining the role of the IG20 gene in ovarian cancer revealed that *MADD* is necessary for malignant cell survival compared to the other three splice variants in PA-1 ovarian carcinoma cells [111]. A deeper understanding of the role that IG20 gene splice variants play in ovarian cancer may reveal their potential therapeutic applicability in treating and overcoming drug resistance.

The protein cFLIP (also known as *CFLAR*, *FLIP*, or *CFLICE*) was identified after the discovery of viral FLIP [120]. Initial studies showed that the gene may have evolved through replication, especially due to its structural homology to caspase-8 [120]. While 11 splice variants for *cFLIP* are expressed on the mRNA level, only three of the isoforms have been expressed as proteins. These proteins include the short isoform, *c-FLIP*_s_, the long isoform, *c-FLIP*_L_, and the short murine isoform, *c-FLIP*_R_, with molecular weights between 20 and 60 kDa [121,122,123]. The mechanism of action and inhibition of cell death by *c-FLIP* has not been clarified; however, studies have revealed that *c-FLIP* interacts with Fas-associated death domain protein and caspase-3, -8, and -10 [121,124,125,126,127,128]. *c-FLIP* is recruited to the death-inducing silencing complex by its death effector domains, which inhibits caspase-8 activation [129,130]. Overexpression of *c-FLIP* has been found in several cancers, including prostate, colorectal, bladder, gastric, breast, and ovarian [131,132,133,134,135,136,137,138]. In colorectal cancer, the overexpression of *cFLIP_L_* in patients was correlated with a lower survival rate due to the fact that *cFLIP* provides protection from apoptosis [133]. Previous studies have shown that the downregulation of *c-FLIP* triggers TRAIL-induced apoptosis in cancers resistant to TRAIL therapy; however, few studies have examined *c-FLIP* expression and knockdown in platinum- and taxane-resistant cancers [139,140,141,142]. Treatment with anti-*cFLIP* mediators in combination with anticancer agents such as doxorubicin, cisplatin, and taxol has been shown to reduce the levels of *c-FLIP* and sensitize cells to chemotherapeutic-mediated apoptosis in human glioma, melanoma, prostate, leukemic, and breast cancer cell lines, demonstrating the potential of *c-FLIP* as a therapeutic target in ovarian cancer [143,144,145].

## 4. DNA Damage and Repair

**Figure 3:**
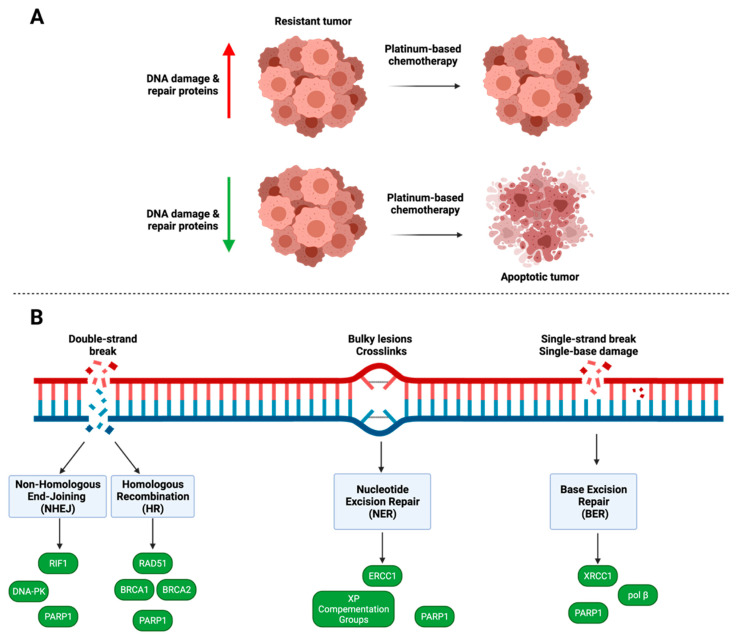
(**A**) Increased expression of DNA damage and repair proteins can cause resistance to platinum-based chemotherapeutics in ovarian cancer. (**B**) DNA damage by chemotherapeutics results in the activation of four DNA repair pathways. Increased expression of proteins within these pathways (green) can reverse this damage through repair mechanisms.

DNA repair pathways play a significant role in cancer drug resistance. First-line chemotherapeutics used in the treatment of ovarian cancer, including platinum-containing drugs, cisplatin and carboplatin, interact with DNA by inducing damage through the formation of DNA adducts [146]. The presence of DNA damage invokes the DNA damage response, a kinase-signaling pathway involved in recognition of damage to DNA structures [146,147]. As DNA repair mechanisms are essential to cell survival, mutations in the DNA damage response have been illustrated to play a significant role in the progression of many types of cancer, including ovarian cancer [147]. Dysfunction of four main DNA repair pathways, including homologous recombination (HR), non-homologous end joining (NHEJ), nucleotide excision repair (NER), and base excision repair (BER), contribute to drug sensitivity, or lack thereof, in ovarian cancer due to the increased expression of genes within these pathways (Figure 3; Table 1) [146]. The downregulation of genes involved in DNA repair pathways has the potential to increase sensitivity to chemotherapeutics for the treatment of ovarian cancer.

### 4.1. PARP1

The poly(ADP-ribose) polymerase (PARP) enzyme family is composed of 17 members which use NAD+ to produce an ADP-ribose posttranslational modification of proteins [151]. *PARP1* is the most well-studied member of the PARP family, and due to its widespread involvement in DNA damage response, many studies implicate the gene in chemotherapeutic resistance in ovarian cancer [154]. Additionally, *PARP1* has activity in more than one DNA repair pathway, where it is involved in the recruitment of repair factors, sensing DNA damage, and coordinating repair [151,179]. In ovarian and breast cancers with a breast cancer type-1 (*BRCA1*) and/or a breast cancer type-2 (*BRCA2*) mutation, PARP inhibitors are currently being used as a therapeutic treatment [149,150,151,154]. PARP inhibitors are typically small-molecule cancer drugs that target *PARP1*’s catalytic activity, causing entrapment at DNA damage sites and blocking BER [179]. In HR repair-deficient cancers, which can result from *BRCA1* and/or *BRCA2* mutations, DNA damage cannot be repaired by either HR or the BER pathway, resulting in cell death [149]. Hegan et al. demonstrated that downregulation of *PARP1* using small molecule inhibitors or siRNA resulted in the decreased expression of both *BRCA1* and *RAD51*, two essential components in the HR pathway in various cancer types [148]. These studies demonstrate that inhibiting *PARP1* is a promising strategy to enhance the efficacy of platinum-based chemotherapeutics, as DNA adducts are mainly repaired through the HR pathway. Additionally, *PARP1* plays a role in the non-homologous end-joining pathway, where coordination between the HR and NHEJ pathways is essential for maintaining genomic stability [157]. Patel et al. examined the role of *PARP1* in NHEJ and whether the inhibition of *PARP1* in HR-deficient ovarian cancer cells would lead to dysfunction in the NHEJ DNA repair pathway because HR-deficient cancers cells rely on the NHEJ pathway to repair DNA double-strand breaks [157]. It was demonstrated that PEO1 human ovarian adenocarcinoma cells with disabled NHEJ repair had decreased sensitivity to PARP inhibitors and siRNA [157]. These results indicate the necessity of NHEJ repair when using PARP inhibitors in HR-deficient ovarian cancer. The NHEJ pathway is more error-prone than HR; therefore, when *PARP1* is inhibited, the error-prone activity of the NHEJ pathway is increased and can lead to increased cytotoxicity [157,186]. *PARP1*’s involvement in multiple DNA repair pathways make it an ideal gene target for treatment of drug-resistant ovarian cancer. Further studies regarding additional PARP family members may elucidate other potential gene targets to help reverse drug resistance in ovarian cancer.

### 4.2. Homologous Recombination

Many tumor types, including ovarian cancer tumors, exhibit defects in HR repair, leading to genomic instability [149]. In ovarian cancer, approximately 50% of tumors display defective HR repair [149]. There is evidence that the loss of genomic stability may generate further mutations, leading to cancer progression [149]. The tumor suppressor genes *BRCA1* and *BRCA2* play significant roles in successful HR [147,149,150]. When DNA damage occurs, *BRCA1* is recruited by *PARP1* and mobilized to the DNA damage site, where the protein becomes part of the *BRCA1*-associated genome surveillance complex [147,149,150,151]. *BRCA2* is more directly involved in repair by regulating *RAD51* recombinase, which binds to the exposed DNA strand [150].

Platinum chemotherapy agents induce DNA double-strand breaks and crosslinks that are repaired through HR and NHEJ [150]. *BRCA1-* and *BRCA2*-deficient ovarian tumors are sensitive to platinum-based chemotherapeutics and small molecule drugs such as PARP inhibitors [149,150]. Therefore, patients with mutated *BRCA1* and *BRCA2* usually have better overall chemotherapy treatment outcomes [149,152]. Zhang et al. determined that increased *BRCA1* expression in epithelial ovarian cancer tumor tissues is associated with resistance to platinum-based drugs, supporting the evidence that loss of *BRCA1* function may contribute to the reversal of resistance [154]. Restoration of *BRCA1* function through the loss of *BRCA1* promoter methylation has been demonstrated to confer resistance to PARP inhibitors in ovarian carcinoma [187]. Additionally, mutation of the *RAD51* binding domain of *BRCA2* has caused HR deficiencies [188]. Labidi-Galy et al. revealed that mutations of the *RAD51* binding domain lead to longer progression-free survival and overall survival in ovarian cancer patients who received platinum-based chemotherapy [188]. Therefore, targeting *BRCA1* and *BRCA2* may reverse acquired resistance in ovarian cancer. However, analysis of acquired PARP inhibitor resistance and its contribution to ovarian cancer progression should be further explored.

Since *BRCA2* is a mediator of *RAD51*, *RAD51*’s role in drug resistance has also been evaluated. When DNA damage occurs, *RAD51* recombinase is transported to the damaged site and loaded onto the damaged strand to help protect the DNA ends from degradation [153]. In triple-negative breast cancer stem cells, Liu et al. found the expression of *RAD51* to be positively correlated with PARP inhibitor insensitivity [155]. After long-term treatment with PARP inhibitors, the triple-negative breast cancer cell lines SUM149 and SUM159 had elevated *RAD51* expression, further confirming that *RAD51* mediates PARP inhibitor resistance; however, knockdown of *RAD51* using short hairpin RNA sensitized the cells to the PARP inhibitor olaparib [155]. While exploring the function of microRNA ler-7e in ovarian cancer, Xiao and colleagues observed that *RAD51* contributes to chemotherapeutic resistance using the chemoresistant epithelial ovarian cancer cell line C13K [156]. *RAD51* protein expression was increased in C13K cells compared to the chemosensitive OV2008 epithelial ovarian cancer cell line and was also associated with decreased survival in patient-derived tissue samples [156].

*RAD51* paralogs, homologous genes that code for proteins with similar functions, are recruited to the site of the damage during DNA strand repair [189]. Deficiencies in *RAD51* paralogs can lead to impaired HR repair and greater sensitivity to platinum-based therapeutics [189]. In the *RAD51* paralog complex, *RAD51*B, *RAD51C,* and *RAD51D* have all been associated with hereditary ovarian and breast cancer [189]. Rivera et al. demonstrated *RAD51D* missense variants resulted in an increased predisposition to high-grade serous ovarian carcinoma in ovarian cancer patients [189]. Additionally, in ovarian cancer patients, the mutations increased sensitivity to PARP inhibitors [189]. While primary *RAD51*D mutations initially sensitize ovarian cancer cells to PARP inhibitors, Kondrashova et al. reported that secondary mutations of both *RAD51*D and *RAD51*C conferred acquired PARP inhibitor resistance similar to the secondary mutations of *BRCA1* or *BRCA2* [190].

### 4.3. Nucleotide Excision Repair

Nucleotide excision repair (NER) is involved in the repair of platinum-induced DNA adducts [167]. Xeroderma Pigmentosa (XP) Complementation Groups A-G are essential genes in the NER pathway, as they are involved in damage recognition, transcription initiation, and stabilization of the damaged DNA strand [167,174]. Although XP genes are typically associated with the hereditary disease of the same name, *XPA*, *XPB*, and *XPF* have been shown to have increased expression in platinum-resistant ovarian cancer cells [167,174]. *XPA* interacts with multiple proteins during DNA repair, including the excision repair cross-complementing group 1-XPF endonuclease, to stabilize the damaged portion of DNA [171,172]. Rosenberg et al. determined that deficient *XPA* expression increased sensitivity to ultraviolet- and platinum-based agents in human non-small lung carcinoma cell lines [176]. While *XPA* is overexpressed in platinum-resistant ovarian cancer tumors, it does not seem to have a role in DNA excision activity [159,173]. *XPB* is directly involved in DNA transcription [159]. Dabholkar et al. detected a five-fold increase in *XPB* mRNA levels in platinum-resistant ovarian tumor tissues compared to platinum-sensitive tissues [175]. A similar increase in expression was observed for both excision repair cross-complementing group 1 (ERCC1) and *XPA* in platinum-resistant ovarian cancer tissues [175]. The *XP* family plays a role in the resistance of ovarian cancer to platinum-based chemotherapeutics; thus, targeting this family may be of therapeutic benefit specifically for the reversal of platinum resistance.

*ERCC1* is a part of the DNA repair endonuclease complex, *ERCC1–XPF* [174]. The *ERCC1–XPF* complex is recruited to damaged sites of DNA through interaction with *XPA* [177]. *ERCC1–XPF* and endonuclease *XPG* cut the damaged strand on the 5′ and 3′ ends, respectively, allowing for repair of the strand [177]. The *ERCC1–XPF* complex and the individual proteins, *XPF* and ERCC1, have been highly studied as cisplatin resistance markers due to the proteins’ involvement in the rate-limiting step of NER [174]. Increased *ERCC1* is correlated with platinum resistance in many cancers, including ovarian, nasopharyngeal, cervical, head and neck squamous carcinoma, lung adenocarcinoma, non-small cell lung cancer, and gastric cancer [174,178]. In ovarian cancer, resistance to platinum-based chemotherapy has been associated with high levels of *ERCC1* mRNA [178]. Through increased exposure of MCAS human ovarian carcinoma cells to cisplatin, Li et al. demonstrated increased mRNA and protein expression of ERCC1 [168]. High expression of *ERCC1* has also been linked to chemoresistance in ovarian cancer patients [154].

While the *ERCC1* protein has its role in DNA repair, the *ERCC1–XPF* endonuclease complex is also implicated in drug resistance. Arora et al. hypothesized that decreased *ERRC1–XPF* in ovarian cancer cells could increase sensitivity to cisplatin [169]. siRNA-mediated knockdown of *ERCC1*, *XPF*, and *ERCC1–XPF* reduced the rate of cisplatin adduct repair in non-small cell lung cancer, ovarian cancer, and breast cancer cell lines. [169]. Decreased levels of *ERCC1–XPF* correlated not only with improved progression-free survival but also increased platinum and PARP inhibitor sensitivity in patient samples of ovarian cancer tissue. [170]. Future studies should determine whether the *ERCC1–XPF* complex can be silenced alone or in addition to ERCC1 and XPF for efficient resensitization to platinum-based chemotherapy. Impairment of NER through the *ERCC1–XPF* heterodimer has therapeutic potential for reducing the cellular capacity to repair platinum-induced DNA damage, allowing for greater sensitivity to platinum-based therapeutics.

### 4.4. Non-Homologous End Joining

Similar to HR, the NHEJ repair pathway repairs double-strand DNA breaks [159]. DNA repair through the NHEJ pathway is induced faster compared to HR, but repair is more error-prone [159]. NHEJ can be split into two sub-pathways, classical and alternative. The classical pathway can function independently of a DNA template, whereas the alternative pathway is only active when HR or the classical pathway is inhibited [159]. Errors in the function of both pathways are associated with drug resistance [159].

Replication timing regulatory factor 1 (*RIF1*) and DNA-dependent protein kinase (*DNA-PK*) are involved in the NHEJ pathway, and both have implications for ovarian cancer drug resistance [158,159]. In the NHEJ pathway, *RIF1* is recruited to DNA double-strand breaks, where the protein blocks double-strand break resection, facilitating DNA repair [158,160,161]. Liu et al. demonstrated that knockdown of *RIF1* resulted in greater cisplatin sensitivity in platinum-sensitive OVCAR3 cells and platinum-resistant A2780 cells [158]. Additionally, analysis of epithelial ovarian cancer tissue revealed that nearly two out of three patients with chemoresistant epithelial ovarian cancer had high expression of *RIF1*, whereas only 34.2% of chemosensitive patients displayed overexpression of *RIF1* [158]. In the HeLa human cervical cancer cell line, *RIF1* knockdown increased cisplatin sensitivity [160]. Because platinum-based chemotherapeutics utilize double-stranded breaks to cause cell death, overexpression of *RIF1* in ovarian cancer would reverse any damage done by chemotherapeutics; therefore, its potential to be a therapeutic target is promising.

*DNA-PK* is a serine/threonine protein kinase that repairs double-strand DNA breaks caused by chemotherapeutic agents and oxidative stress [162,163]. Beyond its role in DNA repair, *DNA-PK* is involved in cell cycle progression, DNA transcription regulation, and telomere maintenance, indicating its vital role in cell survival [163]. *DNA-PK* is also involved in regulating pro-tumorigenic pathways, which promote tumor development, cell survival, and cell proliferation [165]. Increased expression of *DNA-PK* has also been correlated with poor prognosis in ovarian cancer [162,164]. Due to the significant role of *DNA-PK* in the DNA damage response, *DNA-PK* inhibitors, siRNAs, and/or chemical inhibitors have been developed to reduce the ability of cells to perform DNA repair and enhance the efficacy of DNA damaging chemotherapeutics [162,165]. Previous work has specifically examined the use of *DNA-PK* inhibitors or RNAi in reducing chemoresistance in ovarian or breast cancer. Wise et al. revealed that combining *DNA-PK* inhibitors with a platinum-based agent reduces tumor growth in A2780 and SKOV3 ovarian cancer cell lines [162]. A similar study using MDA-MB-231 breast cancer cells demonstrated that downregulation of *DNA-PK* using shRNA resulted in greater cisplatin sensitivity, confirming that *DNA-PK* plays a role in acquired resistance to cisplatin [166]. While these results are promising, few studies use *DNA-PK* as a target in chemoresistant ovarian cancer. More studies targeting *DNA-PK* could further establish its therapeutic benefit.

### 4.5. Base Excision Repair

The base excision repair (BER) pathway is responsible for DNA single-strand break repair as well as removing base lesions caused by alkylating agents such as cisplatin [159]. X-ray repair cross-complementing gene 1 (*XRCC1*) is a 70 kDa molecular scaffold protein that is a critical component of the BER pathway by coordinating DNA repair through interactions with *PARP1* [180]. Overexpression of *XRCC1* in ovarian cancer has previously been associated with platinum-based drug resistance [159,180]. In a study by Abdel-Fatah et al., siRNA-mediated knockdown of *XRCC1* in OVCAR-3 and OVCAR-4 human ovarian cancer cells resulted in greater platinum sensitivity, demonstrating *XRCC1*’s active involvement in platinum resistance in ovarian cancer [180]. In a clinical study, *XRCC1*-positive epithelial ovarian cancer tumors were significantly more likely to be platinum-resistant compared to *XRCC1*-negative tumors [180].

Due to recent studies depicting *XRCC1* as a key component in repairing carboplatin- and cisplatin-induced DNA damage, Zhang et al. evaluated the relationship between *XRRC1* expression and the ability to reverse cisplatin drug resistance [181]. SKOV3/DPP human ovarian cancer cells were treated with heat shock protein 90 inhibitors [181]. Heat shock protein 90 inhibitors have been shown to decrease the stability of many tumor-associated proteins, including *XRCC1* [181]. The results illustrated that decreasing the expression of *XRCC1* using inhibitors of heat shock protein 90 reversed cisplatin resistance in SKOV3/DPP ovarian cancer cells [181]. Similarly, Sawant et al. demonstrated that the downregulation of *XRCC1* in MDA-MB-231 breast cancer cells allowed for more significant cisplatin toxicity [185]. *XRCC1* is an interesting target since few studies have analyzed targeting the protein and its downstream effects. Instead, downregulation of *XRCC1* seems to be due to direct targeting of other proteins. Together, these results indicate the potential of *XRCC1* to be used as a target for drug-resistant ovarian cancer.

Another potential gene target is DNA polymerase β (pol β), the primary polymerase involved in the BER pathway [184]. *Pol β* lacks proofreading capabilities and thus is error-prone [183]. However, high expression and activity levels of *pol β* have been identified in ovarian cancer tumors, and upregulation of *pol β* has been shown to contribute to tumor progression and platinum resistance in many types of cancer, including breast, prostate, and colon cancer [159,182,183]. Little work has been performed exploring *pol β* as a therapeutic target, but the overexpression of the enzyme and its role in tumor progression indicates the therapeutic potential of *pol β* as a target in ovarian cancer.

Because platinum-based chemotherapies cause DNA damage by directly binding to DNA or RNA strands, focusing on overexpressed DNA damage and repair proteins as therapeutic targets could increase sensitivity to chemotherapy. Additionally, it is possible that the dual targeting of genes in different repair pathways could enhance the efficiency of platinum-based drugs, though few studies have investigated this dual approach. Targeting genes involved in platinum-based chemoresistance in the repair pathways, as well as non-repair pathways, may provide effective therapeutic combinations.

## 5. Cancer Stem Cells

**Figure 4:**
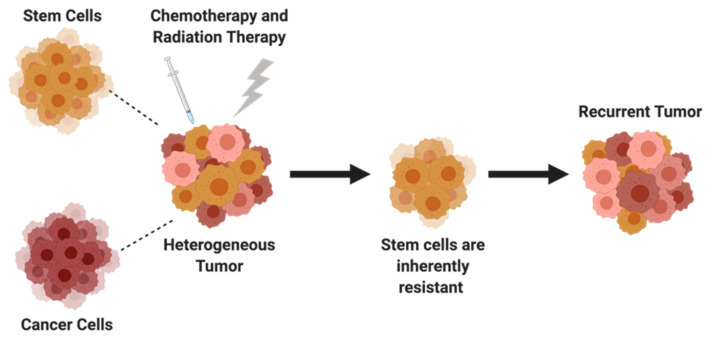
Scheme of cancer stem cells (CSCs) depicting the role of CSCs in ovarian cancer recurrence. CSCs are inherently resistant to chemotherapy and radiation and remain in the tumor tissue after treatment, causing tumor cell production and tumor recurrence. Tumor cells (red), CSCs (yellow).

While the role of chemoresistance has been apparent in drug efflux, apoptosis, and DNA damage and repair, recent studies have investigated distinct subpopulations of cells within ovarian tumors for their potential contribution to drug resistance. The heterogeneity of ovarian tumors makes treatment more difficult because there exists a small subgroup of cancer stem cells (CSCs) or tumor-initiating cells that have been shown to induce chemoresistance and cancer relapse [8]. CSCs are capable of self-renewal, differentiation, and tumorgenicity, and are the driving force behind metastasis and recurrence [191]. Initially, ovarian cancer cells were thought to be chemosensitive before being exposed to therapeutics; however, CSCs are inherently resistant. While conventional chemotherapy can reduce the size of an ovarian tumor, CSCs are not specifically targeted, ultimately leading to disease progression (Figure 4). Therefore, targeted therapy of ovarian CSCs could lead to improved patient survival. The identification and characterization of CSCs are denoted by specific intracellular or cell surface markers such as *CD24*, *CD44*, *CD117*, *CD133*, and aldehyde dehydrogenase. Evidence supporting the identification of these markers has been well described in other reviews [192,193,194]. The development of more effective therapies may require treatments targeting proteins and pathways that promote cancer stem cell growth and survival. The mechanism of chemoresistance caused by CSCs in ovarian cancer is complicated and not fully understood; however, CSCs have slow proliferation rates, a high expression of ATP transporters, and can inactivate cell death pathways [192]. Proteins that have been linked to stemness, chemoresistance, and tumorigenesis but have not been well studied or targeted in ovarian cancer are highlighted.

### 5.1. SOX2, OCT4, NANOG

The transcription factor sex-determining region Y-box 2 (*SOX2*) plays a pivotal role in the maintenance of embryonic stem cells. However, in the last decade, *SOX2* has been characterized beyond its role in embryonic stem cells, and evidence has shown that there may be a therapeutic benefit in targeting *SOX2* to reduce its probable tumor-initiating capacity in various cancers. Research has indicated the involvement of *SOX2* in spheroid formation, drug resistance, growth, and metastasis in several cancers of the breast, stomach, colon, and brain [195,196]. Few studies have critically investigated the role *SOX2* plays in ovarian cancer and its relation to tumor-initiating cells. Elevated *SOX2* gene expression has been identified in ovarian cancer cell lines and patient tissue samples [197,198,199]. However, there is conflicting data regarding the correlation between the expression of *SOX2* and patient prognosis. Increased expression of *SOX2* has been associated with poor prognosis and a higher grade of ovarian cancer [197,200]. Conversely, through the analysis of MDAH-2774 and SKOV3 ovarian cell lines and The Cancer Genome Atlas (TCGA) data sets, Belotte et al. determined that *SOX2* amplification in ovarian cancer leads to improved survival outcomes via a novel p53-dependent mechanism [198]. Thus, the prognostic value of *SOX2* in ovarian cancer must be further investigated. However, a clear connection has been established between increased expression of *SOX2* and chemoresistance, not only in ovarian cancer but also in breast and prostate cancer [195]. In one study, elevated expression of SOX2 in OVCAR3, CAOV3, and OVCAR5 cell lines demonstrated a lack of sensitivity to carboplatin, cisplatin, and paclitaxel [201]. Following the knockdown of *SOX2* using shRNA, the cells exhibited increased sensitivity to chemotherapeutics [201]. Lentiviral re-expression of ectopic *SOX2* reversed chemotherapy sensitivity, demonstrating that *SOX2* may be a molecular driver for chemoresistance in ovarian cancer. Reducing the expression levels of *SOX2* can be therapeutically beneficial for decreasing chemoresistance by decreasing the population of cancer stem cells. Yiping Wen and colleagues investigated this notion using SKOV3 and HO8910 ovarian cells to form spheroids with overexpression of *SOX2*. Knockdown of *SOX2* not only decreased the formation of spheroids, but also reduced the expression of other stemness-related genes and resensitized ovarian cancer spheroids to cisplatin treatment [202].

As a transcription factor, *SOX2* does not work alone but in conjunction with other proteins. Several studies have shown that octamer-binding transcription factor 4 (*OCT4*) and *NANOG* work alongside *SOX2* in a large protein complex amongst other proteins [203,204]. *OCT4* and *NANOG* are significantly overexpressed in poorly differentiated tumors compared to well-differentiated tumors [205]. Increased expression of *OCT4* and *NANOG* is a prognostic factor in several cancers, including breast, colorectal, and ovarian cancer [206,207,208,209]. Numerous studies have also demonstrated that *OCT4* and *NANOG* are associated with chemoresistance in ovarian cancer. One study revealed that ovarian CSCs derived from primary tumors not only had a higher expression of *NANOG* and *OCT4* but were also resistant to treatment with cisplatin and paclitaxel [210]. In another study, paclitaxel-resistant SKOV3 cells had increased expression of *NANOG* in comparison to paclitaxel-sensitive SKOV3 cells, indicating a relationship between *NANOG* expression and resistance [211]. Additionally, knockdown of *OCT4* and *NANOG* in vitro and in vivo using shRNA demonstrated the role both genes played in tumorigenesis, metastasis, and resistance in pancreatic cancer [212]. When silenced, pancreatic stem cells showed increased sensitivity to gemcitabine, a DNA synthesis inhibitor. In addition, knockdown resulted in reduced colony formation and slowed tumor growth when compared to scrambled controls [212]. While studies show that inhibition of *OCT4*- and *NANOG*-associated proteins can reduce their expression, more studies are needed to analyze the effect of direct inhibition of *OCT4* and *NANOG* separately in ovarian cancer.

### 5.2. JAK/STAT Pathway

Aside from regulating cellular processes, the Janus kinase-signal transducer and activator of transcription (JAK-STAT) signaling pathway has been associated with ovarian cancer stemness, cell proliferation, and tumorigenicity [213,214]. A JAK1/2 inhibitor, ruxolitinib, was previously FDA-approved for the treatment of myeloproliferative neoplasms such as myelofibrosis and polycythaemia vera [215]. Currently, ruxolitnib is being repurposed and evaluated as a potential therapeutic option for treating solid cancers such as pancreatic and ovarian cancer. In preclinical studies, ruxolitinib has been shown to sensitize ovarian cancer to paclitaxel [216,217]. Poznansky et al. evaluated the effects of ruxolitinib in in vitro and in vivo ovarian cancer models, revealing that ruxolitinib could resensitize ovarian cancer to taxol at low doses and significantly increase the survival time of diseased mice when treated with a combination of ruxolitinib and taxol [216]. This work was further confirmed when Han et al. investigated whether ruxolitinib could increase the anti-tumor capability of several chemotherapeutics, including paclitaxel, cisplatin, carboplatin, doxorubicin and topotecan [217]. Briefly, Han and colleagues treated human MDAH-2774 and OVCAR-8 ovarian cancer cells with either ruxolitinib, a chemotherapeutic (paclitaxel, cisplatin, carboplatin, doxorubicin, topotecan) or a combination of both, and examined cellular viability. It was demonstrated that a combination of ruxolitinib with chemotherapeutic agents resulted in increased cellular death in comparison to singular treatment of either ruxolitinib or chemotherapeutic alone [217]. Altogether, these studies show that inhibiting the JAK/STAT pathway can increase the sensitivity of resistant ovarian cancer for treatment with chemotherapeutics. While ruxolitinib is an exciting small molecule inhibitor, there are limited studies using gene therapy approaches such as siRNA, which may enhance silencing of JAK or STAT proteins in ovarian cancer and should be explored in future studies.

Specifically targeting genes that promote stem-like characteristics and the survival of CSCs can reduce the likelihood of ovarian tumor recurrence. Silencing cancer stem cell-related genes may even reduce the needed dosage of chemotherapeutics in treating ovarian cancer.

## 6. Clinical Relevance and Future Directions

There are over 150 clinical trials evaluating treatments for resistant or recurrent ovarian cancer. Many clinical trials focus on singular therapy using either a small molecule inhibitor or immunotherapy using a monoclonal antibody to overcome resistance in ovarian cancer. Combination therapy trials utilize small molecule mimetics and inhibitors or monoclonal antibodies followed by traditional chemotherapeutics, such as paclitaxel. These small molecule drugs and monoclonal antibodies typically target specific genes or pathways. However, clinical studies utilizing gene therapies for treating chemoresistance in ovarian cancer are still limited. Gene therapy strategies include replacing mutated tumor suppressor genes, inhibiting oncogenes, suicide gene therapy, genetic immunopotentiation, oncolytic virotherapy, and antiangiogenic gene therapy [218]. Most gene therapy approaches for cancer have yet to be tested in clinical trials despite promising preclinical results. In addition, the few trials using small molecule drugs and monoclonal antibodies to target genes primarily focus on targeting tyrosine kinase receptors such as VEGF for anti-angiogenesis and signal transduction pathways such as AKT for cell survival and growth, as seen in Table 2. Only one potential gene target in this review, *Bcl-2*, is currently being evaluated in clinical trials for resistant ovarian cancer (NCT02591095). This trial uses ABT-263 (navitoclax) as a single agent to inhibit *Bcl-2* and *Bcl-xL*. Another strategy under investigation in clinical trials uses combination therapy, a small molecule inhibitor followed by paclitaxel (NCT02250781). The small molecule, ONC201, causes inactivation of the AKT/ERK signaling pathway by antagonizing the G-coupled receptor DRD2, leading to a reduction in cell proliferation and survival [219,220]. While therapies are being developed to combat resistance, for most of the genes that have been discussed, clinical trials evaluating them as a therapeutic target to treat resistant ovarian cancer are lacking. This suggests that there is a crucial need to assess the downstream effects of more gene targets in ovarian cancer.

One promising therapy, ofranergene obadenovec (VB-111), is currently in Phase III trials for treating resistant/recurrent ovarian cancer. VB-111 is a gene-based anti-cancer therapeutic that uses a dual mechanism approach that targets blood vessels and induces an anti-tumor-directed immune response. VB-111 is comprised of three components: a non-replicating viral vector, a pre-proendothelin promoter, and a Fas-chimera transgene capable of activating the TNFα and Fas pathway to cause apoptosis in endothelial cells. By stimulating death of endothelial cells, angiogenesis is reduced, resulting in tumor starvation and the release of cell debris containing tumor neo-antigens [221]. Antigen-presenting cells ingest neo-antigens which aids in triggering an anti-tumor immune response. While this therapeutic approach does not explicitly target genes related to resistance, it specifically targets blood vessels using genetic engineering. By targeting blood vessels, VB-111 targets tumor vascularity without the development of resistance, attempting to overcome a significant limitation of monoclonal antibodies, proteins, and small molecule inhibitors. Reducing the likelihood of the development of resistance makes VB-111 a sustainable therapeutic for repeated use; however, there remains a critical need to evaluate therapeutic strategies targeting genes and pathways that are specific to resistance.

Gene therapy has the potential to reduce the chance of patients developing resistance by capitalizing on endogenous mechanisms while providing a personalized medicine approach. Gene therapies have several advantages, including safety, high efficacy, and the ability to target proteins and pathways deemed “undruggable.” Co-delivery of RNAi-based gene therapeutics (siRNAs, miRNAs, shRNAs) and chemotherapeutics has become increasingly popular. Typically, a therapy selective to one gene target is utilized to resensitize resistant cells to chemotherapeutics. In clinical trials, there are few studies targeting a gene/pathway with subsequent treatment of a chemotherapeutic (NCT01653912) for the treatment of resistant ovarian cancer. Afuresterib (NCT01653912) is a small orally available drug that inhibits the activity of protein kinase B (protein kinase AKT), which can result in the reduction of cell proliferation and the induction of apoptosis [222]. Afuresterib was administered to patients with resistant ovarian cancer by repeated treatment every three weeks in combination with paclitaxel and carboplatin. The combination had a response rate of over 30%, which compares favorably to the standard of care, platinum-based monotherapy. While this combination has served as a somewhat effective strategy, its therapeutic potential may be limited because drug resistance is multifactorial. Combination delivery by targeting multiple pathways may be more therapeutically potent. Multiple siRNAs can be delivered simultaneously to cancer cells to knockdown several target genes. In a preclinical study, the reduction of *ABCB1* and *BCL-2* proteins via codelivery of siABCB1 and siBCL2, respectively, sensitized paclitaxel- and cisplatin-resistant SKOV3 and A2780 ovarian cancer cells to therapeutics [223]. Knockdown demonstrated the necessity of targeting both genes for enhanced cytotoxicity. When silencing *MDR1* or *BCL-2* separately in paclitaxel-resistant SKOV3 cells, apoptosis or necrosis was observed in 62.7% and 45.6% of cells, respectively. However, when treated with the combination of siMDR1 and siBCL2, 82.2% of cells were apoptotic or necrotic [223], demonstrating that targeting two genes with varying functions can enhance the sensitization of cells to therapeutics more effectively than targeting a single gene. Targeting multiple therapeutic pathways at once is an advantage of RNAi-based strategies and could provide a promising approach to tackling resistance.

Gene editing can also serve as a therapeutic tool for reducing chemoresistance by knocking in or knocking out associated genes. Gene editing as an approach to overcome drug resistance has been thoroughly reviewed elsewhere [224]. Briefly, total removal of a gene correlated to resistance would provide more of a long-term response in comparison to RNAi-based therapies and has been shown to provide therapeutic benefits in ovarian cancer. For example, using the gene editing tool CRISPR/Cas9 to “knock down” ABCB1 can enhance doxorubicin sensitivity in doxorubicin-resistant A2780/ADR human ovarian cancer cells [225]. However, translating these results into animal models would provide more information on the robustness of targeting ABCB1 via gene editing. Once target effects from CRISPR/Cas9 are elucidated, gene editing can be widely used to knock out multiple genes that are solely related to chemoresistance. However, with this approach, CRISPR/Cas9 must be specifically targeted to chemoresistant cells and not healthy cells to reduce any adverse effects of nonspecific delivery and editing in off-target sites.

In addition to directly editing ovarian cancer cells, immune cells that circulate in the bloodstream can be genetically engineered to attack cancer cells. Chimeric antigen receptor-modified T (CAR-T) cells can be utilized as a cell-based gene therapy where a patient’s T cells (immune cells) are genetically engineered to express a chimeric antigen receptor. The CAR-T cells are then able to attack cancer cells by binding to antigens (or receptors) on cancer cells [226,227]. These receptors can be overexpressed receptors on cancer cells. CAR-T therapy has been widely used in hematological malignancies and is currently being explored as a treatment option for ovarian cancer [226,227]. For the treatment of resistant ovarian cancer, there is a limited number of CAR-T therapies in current clinical trials. While CAR-T therapy is a new and exciting treatment strategy, several challenges exist, especially for solid tumors such as ovarian cancer. Namely, selecting a tumor-associated antigen that is only expressed on ovarian cancer cells and is expressed on the majority of ovarian cancer cells is difficult [228]. Additionally, CAR-T therapies can have difficulty extravasating the tumor vasculature, which is why it is more commonly used in hematological malignancies [229]. Therefore, using CAR-T therapy in combination with other gene therapy strategies may limit some of the associated side effects and toxicities. Though T cells are typically used for CAR-T therapy, other immune cells, such as dendritic and natural killer cells, can also be utilized.

Ovarian cancer is a complex, multifactorial disease. Although targeting one gene or pathway may sensitize cells to chemotherapeutics, treating this aggressive disease will take a multipronged approach. It is imperative that increased efforts are put towardcombination approaches. By targeting genes from two different pathways, synergistic effects may be seen and can provide increased sensitivity to chemotherapeutics compared to targeting one gene/pathway alone. Additionally, gene therapy is not selective to only the tumor. Because advanced ovarian cancer metastasizes within the intraperitoneal niche, it is surrounded by a plethora of cells within the tumor microenvironment. Gene therapy can be used to target the cells that interact with the cancerous cells in the tumor niche, such as immune cells. Additional studies should also focus on the combination of tumor targeting and immune cell gene therapy for the treatment of chemoresistant ovarian cancer.

## 7. Conclusions

The development of drug resistance in ovarian cancer is closely related to worsened clinical prognosis and drastically limits the efficacy of current anticancer treatments. Accumulating evidence demonstrates that overexpressed proteins and alterations to signaling pathways lead to chemoresistant ovarian cancer. Genes associated with drug resistance affect different cellular processes, such as drug efflux, apoptosis, and DNA damage and repair. Additionally, CSCs in ovarian tumor tissue contribute to chemoresistance by persisting even after treatment with chemotherapeutics.

There is a need for emerging therapies that utilize genetic engineering for high-precision therapy to treat resistant ovarian cancer. Proteins involved in drug efflux, intrinsic and extrinsic apoptosis, and DNA damage and repair can be beneficial for singular or combinatorial therapy. Current treatments for ovarian cancer could be improved using targeted gene therapies, especially since tumors are heterogeneous, thus maximizing patient response and survival. Targeting genes using RNAi is a promising anticancer strategy seen in preclinical studies because of its ability to reduce the expression of oncoproteins linked to chemoresistance that may otherwise be deemed ‘undruggable.’ Drug-resistant ovarian cancer is a complicated disease; however, current treatment strategies do not address the multifactorial aspects of the disease. Considering the heterogeneity of ovarian tumors, and targeting multiple pathways and proteins may help improve treatment efficacy and outcomes for patients with ovarian cancer.

## Figures and Tables

**Table 1:** Summary of DNA Damage and Repair Proteins Overexpressed in Ovarian Cancer.

DNA Repair Pathway	Overexpressed Protein(s)	References
Homologous Recombination	*RAD51* and *RAD51* paralogs; *BRCA1*; *BRCA2*; *PARP1*	[147,148,149,150,151,152,153,154,155,156]
Non-homologous End Joining	*RIF1*; *DNA-PK*; *PARP1*	[157,158,159,160,161,162,163,164,165,166]
Nucleotide Excision Repair	XP groups *ERCC1*; *PARP1*	[159,167,168,169,170,171,172,173,174,175,176,177,178]
Base Excision Repair	*XRCC1*; *pol β*; *PARP1*	[149,159,179,180,181,182,183,184,185]

**Table 2:** Therapies in clinical trials for the treatment of drug-resistant/recurrent ovarian cancer in the US.

Therapeutic	Target	Drug Type	Phase	Clinical Trial Identifier
ABT-263 (Navitoclax)	*Bcl-XL, Bcl-2*	Small molecule mimetic	II	NCT02591095
GDC-0068 (Ipatasertib)	*AKT1/2/3*	Small molecule inhibitor	II	NCT04561817
Tivozanib	*VEGF*	Small molecule inhibitor	II	NCT01853644
XL999	*VEGFR PDGFR* *FGFR, FLT-3, Src*	Small molecule inhibitor	II	NCT00277290
MM-121 (seribantumab)	HER3 Pathway	Monoclonal antibody	II	NCT01447706
VB-111 (ofranergene obadenovec)	*TNFR1, FAS*	Gene therapy (chimeric gene)	III	NCT03398655
ONC201	*Akt/ERK*	Small molecule inhibitor	II	NCT04055649
ZN-c3	*Wee1*	Small molecule inhibitor	I	NCT05198804
ACR-368	*CHK1/2*	Small molecule inhibitor	I	NCT05548296
Navicixizumab	*DLL4, VEGF*	Monoclonal antibody	III	NCT05043402
Bevacizumab	*VEGF*	Monoclonal antibody	II	NCT05325229
JPI-547	*PARP1/2, TNKS*	Small molecule inhibitor	II	NCT05475184

## Data Availability

Not applicable.

## References

[1] Chandra A., Pius C., Nabeel M., Nair M., Vishwanatha J.K., Ahmad S., Basha R. (2019). Ovarian Cancer: Current Status and Strategies for Improving Therapeutic Outcomes. Cancer Med..

[2] Stewart C., Ralyea C., Lockwood S. (2019). Ovarian Cancer: An Integrated Review. Seminars Oncology Nursing.

[3] (2022). American Cancer Society Key Statistics for Ovarian Cancer. https://www.cancer.org/cancer/ovarian-cancer/about/key-statistics.html.

[4] Ovarian Cancer—Cancer Stat Facts (2022). National Cancer Institute: Surveillance, Epidemiology, and End Results Program. https://seer.cancer.gov/statfacts/html/ovary.html.

[5] Cortez A.J., Tudrej P., Kujawa K.A., Lisowska K.M. (2018). Advances in Ovarian Cancer Therapy. Cancer Chemother. Pharmacol..

[6] Brasseur K., Gévry N., Asselin E. (2017). Chemoresistance and Targeted Therapies in Ovarian and Endometrial Cancers. Oncotarget.

[7] Cornelison R., Llaneza D.C., Landen C.N. (2017). Emerging Therapeutics to Overcome Chemoresistance in Epithelial Ovarian Cancer: A Mini-Review. Int. J. Mol. Sci..

[8] Holohan C., Van Schaeybroeck S., Longley D.B., Johnston P.G. (2013). Cancer Drug Resistance: An Evolving Paradigm. Nat. Rev. Cancer.

[9] Gatti L., Zunino F. (2005). Overview of Tumor Cell Chemoresistance Mechanisms. Methods Mol. Med..

[10] Nikolaou M., Pavlopoulou A., Georgakilas A.G., Kyrodimos E. (2018). The Challenge of Drug Resistance in Cancer Treatment: A Current Overview. Clin. Exp. Metastasis.

[11] Dean M., Allikmets R. (2001). Complete Characterization of the Human ABC Gene Family. J. Bioenerg. Biomembr..

[12] Dean M., Hamon Y., Chimini G. (2001). The Human ATP-Binding Cassette (ABC) Transporter Superfamily. J. Lipid Res..

[13] Robey R.W., Pluchino K.M., Hall M.D., Fojo A.T., Bates S.E., Gottesman M.M. (2018). Revisiting the Role of ABC Transporters in Multidrug-Resistant Cancer. Nat. Rev. Cancer.

[14] Lage H. (2006). MDR1/P-Glycoprotein (ABCB1) as Target for RNA Interference-Mediated Reversal of Multidrug Resistance. Curr. Drug Targets.

[15] Huang Y., Sadée W. (2006). Membrane Transporters and Channels in Chemoresistance and -Sensitivity of Tumor Cells. Cancer Lett..

[16] Freimund A.E., Beach J.A., Christie E.L., Bowtell D.D.L. (2018). Mechanisms of Drug Resistance in High-Grade Serous Ovarian Cancer. Hematol. Oncol. Clin. North Am..

[17] Ren F., Shen J., Shi H., Hornicek F.J., Kan Q., Duan Z. (2016). Novel Mechanisms and Approaches to Overcome Multidrug Resistance in the Treatment of Ovarian Cancer. Biochim. Biophys. Acta Rev. Cancer.

[18] Choi C.H. (2005). ABC Transporters as Multidrug Resistance Mechanisms and the Development of Chemosensitizers for Their Reversal. Cancer Cell Int..

[19] Penson R.T., Oliva E., Skates S.J., Glyptis T., Fuller A.F., Goodman A., Seiden M.V. (2004). Expression of Multidrug Resistance-1 Protein Inversely Correlates with Paclitaxel Response and Survival in Ovarian Cancer Patients: A Study in Serial Samples. Gynecol. Oncol..

[20] Hille S., Rein D.T., Riffelmann M., Neumann R., Sartorius J., Pfützner A., Kurbacher C.M., Schöndorf T., Breidenbach M. (2006). Anticancer Drugs Induce Mdr1 Gene Expression in Recurrent Ovarian Cancer. Anticancer Drugs.

[21] Masanek U., Stammler G., Volm M. (1997). Messenger RNA Expression of Resistance Proteins and Related Factors in Human Ovarian Carcinoma Cell Lines Resistant to Doxorubicin, Taxol and Cisplatin. Anticancer Drugs.

[22] Pan J., Mendes L.P., Yao M., Filipczak N., Garai S., Thakur G.A., Sarisozen C., Torchilin V.P. (2019). Polyamidoamine Dendrimers-Based Nanomedicine for Combination Therapy with SiRNA and Chemotherapeutics to Overcome Multidrug Resistance. Eur. J. Pharm. Biopharm..

[23] Yang X., Lyer A.K., Singh A., Choy E., Hornicek F.J., Amiji M.M., Duan Z. (2015). MDR1 SiRNA Loaded Hyaluronic Acid-Based CD44 Targeted Nanoparticle Systems Circumvent Paclitaxel Resistance in Ovarian Cancer. Sci. Rep..

[24] Rosenberg M.F., Mao Q., Holzenburg A., Ford R.C., Deeley R.G., Cole S.P.C. (2001). The Structure of the Multidrug Resistance Protein 1 (MRP1/ABCC1): Crystallization and Single-Particle Analysis. J. Biol. Chem..

[25] Bakos É., Homolya L. (2007). Portrait of Multifaceted Transporter, the Multidrug Resistance-Associated Protein 1 (MRP1/ABCC1). Pflug. Arch. Eur. J. Physiol..

[26] Almquist K.C., Loe D.W., Hipfner D.R., Mackie J.E., Cole S.P.C., Deeley R.G. (1995). Characterization of the Mr 190,000 Multidrug Resistance Protein (MRP) in Drug-Selected and Transfected Human Tumor Cells. Cancer Res..

[27] Cole S.P.C., Bhardwaj G., Gerlach J.H., Mackie J.E., Grant C.E., Almquist K.C., Stewart A.J., Kurz E.U., Duncan A.M.V., Deeley R.G. (1992). Overexpression of a Transporter Gene in a Multidrug-Resistant Human Lung Cancer Cell Line. Science.

[28] Tong X., Zhao J., Zhang Y., Mu P., Wang X. (2019). Expression Levels of MRP1, GST-π, and GSK3Β in Ovarian Cancer and the Relationship with Drug Resistance and Prognosis of Patients. Oncol. Lett..

[29] Kavallaris M., Leary J.A., Barrett J.A., Friedlander M.L. (1996). MDR1 and Multidrug Resistance-Associated Protein (MRP) Gene Expression in Epithelial Ovarian Tumors. Cancer Lett..

[30] Ohishi Y., Oda Y., Uchiumi T., Kobayashi H., Hirakawa T., Miyamoto S., Kinukawa N., Nakano H., Kuwano M., Tsuneyoshi M. (2002). ATP-Binding Cassette Superfamily Transporter Gene Expression in Human Primary Ovarian Carcinoma. Clin. Cancer Res..

[31] Ehrlichova M., Mohelnikova-Duchonova B., Hrdy J., Brynychova V., Mrhalova M., Kodet R., Rob L., Pluta M., Gut I., Soucek P. (2013). The Association of Taxane Resistance Genes with the Clinical Course of Ovarian Carcinoma. Genomics.

[32] Tong W.Y., Alnakhli M., Bhardwaj R., Apostolou S., Sinha S., Fraser C., Kuchel T., Kuss B., Voelcker N.H. (2018). Delivery of SiRNA in Vitro and in Vivo Using PEI-Capped Porous Silicon Nanoparticles to Silence MRP1 and Inhibit Proliferation in Glioblastoma. J. Nanobiotechnol..

[33] Tivnan A., Zakaria Z., O’Leary C., Kögel D., Pokorny J.L., Sarkaria J.N., Prehn J.H.M. (2015). Inhibition of Multidrug Resistance Protein 1 (MRP1) Improves Chemotherapy Drug Response in Primary and Recurrent Glioblastoma Multiforme. Front. Neurosci..

[34] Shao S.L., Cui T.T., Zhao W., Zhang W.W., Xie Z.L., Wang C.H., Jia H.S., Liu Q. (2014). RNAi-Based Knockdown of Multidrug Resistance-Associated Protein 1 Is Sufficient to Reverse Multidrug Resistance of Human Lung Cells. Asian Pac. J. Cancer Prev..

[35] Chen N., Kong Y., Wu Y., Gao Q., Fu J., Sun X., Geng Q. (2019). CAC1 Knockdown Reverses Drug Resistance through the Downregulation of P-Gp and MRP-1 Expression in Colorectal Cancer. PLoS ONE.

[36] Gana C.C., Hanssen K.M., Yu D.M.T., Flemming C.L., Wheatley M.S., Conseil G., Cole S.P.C., Norris M.D., Haber M., Fletcher J.I. (2019). MRP1 Modulators Synergize with Buthionine Sulfoximine to Exploit Collateral Sensitivity and Selectively Kill MRP1-Expressing Cancer Cells. Biochem. Pharmacol..

[37] Schinkel A.H., Jonker J.W. (2003). Mammalian Drug Efflux Transporters of the ATP Binding Cassette (ABC) Family: An Overview. Adv. Drug Deliv. Rev..

[38] Natarajan K., Xie Y., Baer M.R., Ross D.D. (2012). Role of Breast Cancer Resistance Protein (BCRP/ABCG2) in Cancer Drug Resistance. Biochem. Pharmacol..

[39] Austin Doyle L., Yang W., Abruzzo L.V., Krogmann T., Gao Y., Rishi A.K., Ross D.D. (1998). A Multidrug Resistance Transporter from Human MCF-7 Breast Cancer Cells. Proc. Natl. Acad. Sci. USA.

[40] Messenger P., Expression R.N.A., Ross D.D., Yang W., Abruzzo L.V., Dalton W.S., Lage H., Dietel M., Greenberger L., Cole S.P.C. (1999). Atypical Multidrug Resistance: Breast Cancer Resistance. J. Natl. Cancer Inst..

[41] Gottesman M.M., Fojo T., Bates S.E. (2002). Multidrug Resistance in Cancer: Role of ATP-Dependent Transporters. Nat. Rev. Cancer.

[42] Zahreddine H., Borden K.L.B. (2013). Mechanisms and Insights into Drug Resistance in Cancer. Front. Pharmacol..

[43] Maliepaard M., Van Gastelen A., De Jong L.A., Pluim D., Van Waardenburg R.C.A.M., Ruevekamp-Helmers M.C., Floot B.G.J., Schellens J.H.M. (1999). Advances in Brief Overexpression of the BCRP/MXR/ABCP Gene in a Topotecan-Selected Ovarian. Cancer Res..

[44] Januchowski R., Sterzyńska K., Zaorska K., Sosińska P., Klejewski A., Brązert M., Nowicki M., Zabel M. (2016). Analysis of MDR Genes Expression and Cross-Resistance in Eight Drug Resistant Ovarian Cancer Cell Lines. J. Ovarian Res..

[45] Mo L., Pospichalova V., Huang Z., Murphy S.K., Payne S., Wang F., Kennedy M., Cianciolo G.J., Bryja V., Pizzo S.V. (2015). Ascites Increases Expression/Function of Multidrug Resistance Proteins in Ovarian Cancer Cells. PLoS ONE.

[46] Ricci J.W., Lovato D.M., Severns V., Sklar L.A., Richard S. (2017). Novel ABCG2 Antagonists Reverse Topotecan-Mediated Chemotherapeutic Resistance in Ovarian Carcinoma Xenografts. Mol. Cancer Ther..

[47] Kaufmann S.H., Earnshaw W.C. (2000). Induction of Apoptosis by Cancer Chemotherapy. Exp. Cell Res..

[48] Pistritto G., Trisciuoglio D., Ceci C., Garufi A., D’Orazi G. (2016). Apoptosis as Anticancer Mechanism: Function and Dysfunction of Its Modulators and Targeted Therapeutic Strategies. Aging.

[49] Binju M., Amaya-Padilla M.A., Wan G., Gunosewoyo H., Rahmanto Y.S., Yu Y. (2019). Therapeutic Inducers of Apoptosis in Ovarian Cancer. Cancers.

[50] Pfeffer C.M., Singh A.T.K. (2018). Apoptosis: A Target for Anticancer Therapy. Int. J. Mol. Sci..

[51] Li J., Yuan J. (2008). Caspases in Apoptosis and Beyond. Oncogene.

[52] Kim M., Hernandez L., Annunziata C.M. (2016). Caspase 8 Expression May Determine the Survival of Women with Ovarian Cancer. Cell Death Dis..

[53] Cory S., Adams J.M. (2002). The BCL2 Family: Regulators of the Cellular Life-or-Death Switch. Nat. Rev. Cancer.

[54] Maji S., Panda S., Samal S.K., Shriwas O., Rath R., Pellecchia M., Emdad L., Das S.K., Fisher P.B., Dash R. (2018). Bcl-2 Antiapoptotic Family Proteins and Chemoresistance in Cancer.

[55] Villedieu M., Louis M.H., Dutoit S., Brotin E., Lincet H., Duigou F., Staedel C., Gauduchon P., Poulain L. (2007). Absence of Bcl-XL down-Regulation in Response to Cisplatin Is Associated with Chemoresistance in Ovarian Carcinoma Cells. Gynecol. Oncol..

[56] Brotin E., Meryet-Figuière M., Simonin K., Duval R.E., Villedieu M., Leroy-Dudal J., Saison-Behmoaras E., Gauduchon P., Denoyelle C., Poulain L. (2010). Bcl-XL and MCL-1 Constitute Pertinent Targets in Ovarian Carcinoma and Their Concomitant Inhibition Is Sufficient to Induce Apoptosis. Int. J. Cancer.

[57] Williams J., Lucas P.C., Griffith K.A., Choi M., Fogoros S., Hu Y.Y., Liu J.R. (2005). Expression of Bcl-XL in Ovarian Carcinoma Is Associated with Chemoresistance and Recurrent Disease. Gynecol. Oncol..

[58] Mano Y., Kikuchi Y., Yamamoto K., Kita T., Hirata J., Tode T., Ishii K., Nagata I. (1999). Bcl-2 as a Predictor of Chemosensitivity and Prognosis in Primary Epithelial Ovarian Cancer. Eur. J. Cancer.

[59] Yang Y., Li S., Sun Y., Zhang D., Zhao Z., Liu L. (2019). Reversing Platinum Resistance in Ovarian Cancer Multicellular Spheroids by Targeting Bcl-2. Onco. Targets. Ther..

[60] Liu J.R., Fletcher B., Page C., Hu C., Nunez G., Baker V. (1998). Bcl-x(L) Is Expressed in Ovarian Carcinoma and Modulates Chemotherapy- Induced Apoptosis. Gynecol. Oncol..

[61] Igney F.H., Krammer P.H. (2002). Death and Anti-Death: Tumour Resistance to Apoptosis. Nat. Rev. Cancer.

[62] Fulda S. (2015). Targeting Extrinsic Apoptosis in Cancer: Challenges and Opportunities. Semin. Cell Dev. Biol..

[63] Kim R. (2005). Recent Advances in Understanding the Cell Death Pathways Activated by Anticancer Therapy. Cancer.

[64] Du C., Fang M., Li Y., Li L., Wang X. (2000). Smac, a Mitochondrial Protein That Promotes Cytochrome c-Dependent Caspase Activation by Eliminating IAP Inhibition. Cell.

[65] Al-Alem L.F., Baker A.T., Pandya U.M., Eisenhauer E.L., Rueda B.R. (2019). Understand and Targeting Apoptotic Pathways in Ovarian Cancer. Cancers.

[66] Hassan M., Watari H., Abualmaaty A., Ohba Y., Sakuragi N. (2014). Apoptosis and Molecular Targeting Therapy in Cancer. Biomed. Res. Int..

[67] Cheng J.Q., Jiang X., Fraser M., Li M., Dan H.C., Sun M., Tsang B.K. (2002). Role of X-Linked Inhibitor of Apoptosis Protein in Chemoresistance in Ovarian Cancer: Possible Involvement of the Phosphoinositide-3 Kinase/Akt Pathway. Drug Resist. Updat..

[68] Sapi E., Alvero A.B., Chen W., O’Malley D., Hao X.Y., Dwipoyono B., Garg M., Kamsteeg M., Rutherford T., Mor G. (2004). Resistance of Ovarian Carcinoma Cells to Docetaxel Is XIAP Dependent and Reversible by Phenoxodiol. Oncol. Res..

[69] Asselin E., Mills G.B., Tsang B.K. (2001). XIAP Regulates Akt Activity and Caspase-3-Dependent Cleavage during Cisplatin-Induced Apoptosis in Human Ovarian Epithelial Cancer Cells. Cancer Res..

[70] Zhang Y., Huang F., Luo Q., Wu X., Liu Z., Chen H., Huang Y. (2018). Inhibition of XIAP Increases Carboplatin Sensitivity in Ovarian Cancer. Onco. Targets. Ther..

[71] Miyamoto M., Takano M., Iwaya K., Shinomiya N., Kato M., Aoyama T., Sasaki N., Goto T., Suzuki A., Hitrata J. (2014). X-Chromosome-Linked Inhibitor of Apoptosis as a Key Factor for Chemoresistance in Clear Cell Carcinoma of the Ovary. Br. J. Cancer.

[72] Ma J.J., Chen B.L., Xin X.Y. (2009). XIAP Gene Downregulation by Small Interfering RNA Inhibits Proliferation, Induces Apoptosis, and Reverses the Cisplatin Resistance of Ovarian Carcinoma. Eur. J. Obstet. Gynecol. Reprod. Biol..

[73] Cossu F., Milani M., Mastrangelo E., Lecis D. (2019). Targeting the BIR Domains of Inhibitor of Apoptosis (IAP) Proteins in Cancer Treatment. Comput. Struct. Biotechnol. J..

[74] Cho H.J., Kim H.R., Park Y.S., Kim Y.H., Kim D.K., Park S. (2015). Il Prognostic Value of Survivin Expression in Stage III Non-Small Cell Lung Cancer Patients Treated with Platinum-Based Therapy. Surg. Oncol..

[75] Ambrosini G., Adida C., Altieri D.C. (1997). A Novel Anti-Apoptosis Gene, Survivin, Expressed in Cancer and Lymphoma. Nat. Med..

[76] Ai Z., Yin L., Zhou X., Zhu Y., Zhu D., Yu Y., Feng Y. (2006). Inhibition of Survivin Reduces Cell Proliferation and Induces Apoptosis in Human Endometrial Cancer. Cancer.

[77] Yang D., Welm A., Bishop J.M. (2004). Cell Division and Cell Survival in the Absence of Survivin. Proc. Natl. Acad. Sci. USA.

[78] Altieri D.C. (2003). Validating Survivin as a Cancer Therapeutic Target. Nat. Rev. Cancer.

[79] Mita A.C., Mita M.M., Nawrocki S.T., Giles F.J. (2008). Survivin: Key Regulator of Mitosis and Apoptosis and Novel Target for Cancer Therapeutics. Clin. Cancer Res..

[80] Li F., Ackermann E.J., Bennett C.F., Rothermel A.L., Plescia J., Tognin S., Villa A., Marchisio P.C., Altieri D.C. (1999). Pleiotropic Cell-Division Defects and Apoptosis Induced by Interference with Survivin Function. Nat. Cell Biol..

[81] Carvalho A., Carmena M., Sambade C., Earnshaw W.C., Wheatley S.P. (2003). Survivin Is Required for Stable Checkpoint Activation in Taxol-Treated HeLa Cells. J. Cell Sci..

[82] Skoufias D.A., Mollinari C., Lacroix F.B., Margolis R.L. (2000). Human Survivin Is a Kinetochore-Associated Passenger Protein. J. Cell Biol..

[83] Fortugno P., Wall N.R., Giodini A., O’Connor D.S., Plescia J., Padgett K.M., Tognin S., Marchisio P.C., Altieri D.C. (2002). Survivin Exists in Immunochemically Distinct Subcellular Pools and Is Involved in Spindle Microtubule Function. J. Cell Sci..

[84] Li F., Ambrosini G., Chu E.Y., Plescia J., Tognin S., Marchisio P.C., Altieri D.C. (1998). Control of Apoptosis and Mitotic Spindle Checkpoint by Survivin. Nature.

[85] Zaffaroni N., Pennati M., Colella G., Perego P., Supino R., Gatti L., Pilotti S., Zunino F., Daidone M.G. (2002). Expression of the Anti-Apoptotic Gene Survivin Correlates with Taxol Resistance in Human Ovarian Cancer. Cell. Mol. Life Sci..

[86] Kar R., Palanichamy J.K., Banerjee A., Chattopadhyay P., Jain S.K., Singh N. (2015). Survivin SiRNA Increases Sensitivity of Primary Cultures of Ovarian Cancer Cells to Paclitaxel. Clin. Transl. Oncol..

[87] Chandele A., Prasad V., Jagtap J.C., Shukla R., Shastry P.R. (2004). Upregulation of Survivin in G2/M Cells and Inhibition of Caspase 9 Activity Enhances Resistance in Staurosporine-Induced Apoptosis. Neoplasia.

[88] Shin S., Sung B.J., Cho Y.S., Kim H.J., Ha N.C., Hwang J.I., Chung C.W., Jung Y.K., Oh B.H. (2001). An Anti-Apoptotic Protein Human Survivin Is a Direct Inhibitor of Caspase-3 and -7. Biochemistry.

[89] Banks D.P., Plescia J., Altieri D.C., Haven N., Chen J., Rosenberg S.H., Zhang H., Ng S., Laboratories A., Park A. (2000). Survivin Does Not Inhibit Caspase-3 Activity. Blood J. Am. Soc. Hematol..

[90] Conway E.M., Pollefeyt S., Cornelissen J., DeBaere I., Steiner-Mosonyi M., Ong K., Baens M., Collen D., Schuh A.C. (2000). Three Differentially Expressed Survivin CDNA Variants Encode Proteins with Distinct Antiapoptotic Functions. Blood.

[91] Song Z., Yao X., Wu M. (2003). Direct Interaction between Survivin and Smac/DIABLO Is Essential for the Anti-Apoptotic Activity of Survivin during Taxol-Induced Apoptosis. J. Biol. Chem..

[92] Blanc-Brude O.P., Mesri M., Wall N.R., Plescia J., Dohi T., Altieri D.C. (2003). Therapeutic Targeting of the Survivin Pathway in Cancer: Initiation of Mitochondrial Apoptosis and Suppression of Tumor-Associated Angiogenesis. Clin. Cancer Res..

[93] Cohen C., Lohmann C.M., Cotsonis G., Lawson D., Santoianni R. (2003). Survivin Expression in Ovarian Carcinoma: Correlation with Apoptotic Markers and Prognosis. Mod. Pathol..

[94] Sui L., Dong Y., Ohno M., Watanabe Y., Sugimoto K., Tokuda M. (2002). Survivin Expression and Its Correlation with Cell Proliferation and Prognosis in Epithelial Ovarian Tumors. Int. J. Oncol..

[95] Gąsowska-Bodnar A., Bodnar L., Dąbek A., Cichowicz M., Jerzak M., Cierniak S., Kozłowski W., Baranowski W. (2014). Survivin Expression as a Prognostic Factor in Patients with Epithelial Ovarian Cancer or Primary Peritoneal Cancer Treated with Neoadjuvant Chemotherapy. Int. J. Gynecol. Cancer.

[96] Felisiak-Golabek A., Rembiszewska A., Rzepecka I.K., Szafron L., Madry R., Murawska M., Napiorkowski T., Sobiczewski P., Osuch B., Kupryjanczyk J. (2011). Nuclear Survivin Expression Is a Positive Prognostic Factor in Taxane-Platinum-Treated Ovarian Cancer Patients. J. Ovarian Res..

[97] Chen Z., Naito M., Hori S., Mashima T., Yamori T., Tsuruo T. (1999). A Human IAP-Family Gene, Apollon, Expressed in Human Brain Cancer Cells. Biochem. Biophys. Res. Commun..

[98] Hauser H.P., Bardroff M., Pyrowolakis G., Jentsch S. (1998). A Giant Ubiquitin-Conjugating Enzyme Related to IAP Apoptosis Inhibitors. J. Cell Biol..

[99] Hao Y., Sekine K., Kawabata A., Nakamura H., Ishioka T., Ohata H., Katayama R., Hashimoto C., Zhang X., Noda T. (2004). Apollon Ubiquitinates SMAC and Caspase-9, and Has an Essential Cytoprotection Function. Nat. Cell Biol..

[100] Qiu X.B., Goldberg A.L. (2005). The Membrane-Associated Inhibitor of Apoptosis Protein, BRUCE/Apollon, Antagonizes Both the Precursor and Mature Forms of Smac and Caspase-9. J. Biol. Chem..

[101] Low C.G., Luk I.S.U., Lin D., Fazli L., Yang K., Xu Y., Gleave M., Gout P.W., Wang Y. (2013). BIRC6 Protein, an Inhibitor of Apoptosis: Role in Survival of Human Prostate Cancer Cells. PLoS ONE.

[102] Dong X., Lin D., Low C., Vucic E.A., English J.C., Yee J., Murray N., Lam W.L., Ling V., Lam S. (2013). Elevated Expression of Birc6 Protein in Non-Small-Cell Lung Cancers Is Associated with Cancer Recurrence and Chemoresistance. J. Thorac. Oncol..

[103] Hu T., Weng S., Tang W., Xe R., Chen S., Cai G., Cai Y., Shen X., Zhang S., Dong L. (2015). Overexpression of BIRC6 Is a Predictor of Prognosis for Colorectal Cancer. PLoS ONE.

[104] Li R., Chen B.L., Zhou Y.W., Guo R.W., Shuai M.T., Zeng J.X., Leng A.M. (2016). Expression and Clinical Significance of Apollon in Esophageal Squamous Cell Carcinoma. Mol. Med. Rep..

[105] Wang L., Chen Y.J., Hou J., Wang Y.Y., Tang W.Q., Shen X.Z., Tu R.Q. (2014). Expression and Clinical Significance of BIRC6 in Human Epithelial Ovarian Cancer. Tumor Biol..

[106] Lopergolo A., Pennati M., Gandellini P., Orlotti N.I., Poma P., Daidone M.G., Folini M., Zaffaroni N. (2009). Apollon Gene Silencing Induces Apoptosis in Breast Cancer Cells through P53 Stabilisation and Caspase-3 Activation. Br. J. Cancer.

[107] Garrison J.B. (2015). Knockdown of the Inhibitor of Apoptosis BRUCE Sensitizes Resistant Breast Cancer Cells to Chemotherapeutic Agents. J. Cancer Sci. Ther..

[108] Bodmer J.L., Holler N., Reynard S., Vinciguerra P., Schneider P., Juo P., Blenis J., Tschopp J. (2000). TRAIL Receptor-2 Signals Apoptosis through FADD and Caspase-8. Nat. Cell Biol..

[109] Kischkel F.C., Lawrence D.A., Chuntharapai A., Schow P., Kim K.J., Ashkenazi A. (2000). Apo2L/TRAIL-Dependent Recruitment of Endogenous FADD and Caspase-8 to Death Receptors 4 and 5. Immunity.

[110] Zhang L., Fang B. (2005). Mechanisms of Resistance to TRAIL-Induced Apoptosis in Cancer. Cancer Gene Ther..

[111] Mulherkar N., Ramaswamy M., Mordi D.C., Prabhakar B.S. (2006). MADD/DENN Splice Variant of the IG20 Gene Is Necessary and Sufficient for Cancer Cell Survival. Oncogene.

[112] Mulherkar N., Prasad K.V., Prabhakar B.S. (2007). MADD/DENN Splice Variant of the IG20 Gene Is a Negative Regulator of Caspase-8 Activation: Knockdown Enhances Trail-Induced Apoptosis of Cancer Cells. J. Biol. Chem..

[113] Al-Zoubi A.M., Efimova E.V., Kaithamana S., Martinez O., El-Idrissi M.E.A., Dogan R.E., Prabhakar B.S. (2001). Contrasting Effects of IG20 and Its Splice Isoforms, MADD and DENN-SV, on Tumor Necrosis Factor α-Induced Apoptosis and Activation of Caspase-8 and -3. J. Biol. Chem..

[114] Li P., Jayarama S., Ganesh L., Mordi D., Carr R., Kanteti P., Hay N., Prabhakar B.S. (2010). Akt-Phosphorylated Mitogen-Activated Kinase-Activating Death Domain Protein (MADD) Inhibits TRAIL-Induced Apoptosis by Blocking Fas-Associated Death Domain (FADD) Association with Death Receptor 4. J. Biol. Chem..

[115] Saini S., Sripada L., Tulla K., Kumar P., Yue F., Kunda N., Maker A.V., Prabhakar B.S. (2019). Loss of MADD Expression Inhibits Cellular Growth and Metastasis in Anaplastic Thyroid Cancer. Cell Death Dis..

[116] Subramanian M., Pilli T., Bhattacharya P., Pacini F., Nikiforov Y.E., Kanteti P.V., Prabhakar B.S. (2009). Knockdown of IG20 Gene Expression Renders Thyroid Cancer Cells Susceptible to Apoptosis. J. Clin. Endocrinol. Metab..

[117] Turner A., Li L.C., Pilli T., Qian L., Wiley E.L., Setty S., Christov K., Ganesh L., Maker A.V., Li P. (2013). MADD Knock-Down Enhances Doxorubicin and TRAIL Induced Apoptosis in Breast Cancer Cells. PLoS ONE.

[118] Bi W., Wei Y., Wu J., Sun G., Guo Y., Zhang Q., Dong L. (2013). MADD Promotes the Survival of Human Lung Adenocarcinoma Cells by Inhibiting Apoptosis. Oncol. Rep..

[119] Li L.-C., Jayaram S., Ganesh L., Qian L., Rotmensch J., Maker A.V., Prabhakar B.S. (2011). Knockdown of MADD and C-FLIP Overcomes Resistance to TRAIL-Induced Apoptosis in Ovarian Cancer Cells. Am J Obs. Gynecol.

[120] Thome M., Schneider P., Hofmann K., Fickenscher H., Meinl E., Neipel F., Mattmann C., Burns K., Bodmer J.L., Schröter M. (1997). Viral FLICE-Inhibitory Proteins (FLIPs) Prevent Apoptosis Induced by Death Receptors. Nature.

[121] Irmler M., Thome M., Hahne M., Schneider P., Hofmann K., Steiner V., Bodmer J.L., Schröter M., Burns K., Mattmann C. (1997). Inhibition of Death Receptor Signals by Cellular FLIP. Nature.

[122] Golks A., Brenner D., Fritsch C., Krammer P.H., Lavrik I.N. (2005). C-FLIPR, a New Regulator of Death Receptor-Induced Apoptosis. J. Biol. Chem..

[123] Ram D.R., Ilyukha V., Volkova T., Buzdin A., Tai A., Smirnova I., Poltorak A. (2016). Balance between Short and Long Isoforms of CFLIP Regulates Fas-Mediated Apoptosis in Vivo. Proc. Natl. Acad. Sci. USA.

[124] Scaffidi C., Schmitz I., Krammer P.H., Peter M.E. (1999). The Role of C-FLIP in Modulation of CD95-Induced Apoptosis. J. Biol. Chem..

[125] Micheau O., Thome M., Schneider P., Holler N., Tschopp J., Nicholson D.W., Briand C., Grütter M.G. (2002). The Long Form of FLIP Is an Activator of Caspase-8 at the Fas Death-Inducing Signaling Complex. J. Biol. Chem..

[126] Hughes M.A., Powley I.R., Jukes-Jones R., Horn S., Feoktistova M., Fairall L., Schwabe J.W.R., Leverkus M., Cain K., MacFarlane M. (2016). Co-Operative and Hierarchical Binding of c-FLIP and Caspase-8: A Unified Model Defines How c-FLIP Isoforms Differentially Control Cell Fate. Mol. Cell.

[127] Hwang E.Y., Jeong M.S., Park S.Y., Jang S.B. (2014). Evidence of Complex Formation between FADD and C-FLIP Death Effector Domains for the Death Inducing Signaling Complex. BMB Rep..

[128] Hu S., Vincenz C., Ni J., Gentz R., Dixit V.M. (1997). I-FLICE, a Novel Inhibitor of Tumor Necrosis Factor Receptor-1- and CD- 95-Induced Apoptosis. J. Biol. Chem..

[129] Krueger A., Baumann S., Krammer P.H., Kirchhoff S. (2001). FLICE-Inhibitory Proteins: Regulators of Regulators of Death. Mol. Cell. Biol..

[130] Hillert L.K., Ivanisenko N.V., Espe J., König C., Ivanisenko V.A., Kähne T., Lavrik I.N. (2020). Long and Short Isoforms of C-FLIP Act as Control Checkpoints of DED Filament Assembly. Oncogene.

[131] Yun H., Xie J., Olumi A.F., Ghosh R., Kumar A.P. (2015). Activation of AKR1C1/ERβ Induces Apoptosis by Downregulation of c-FLIP in Prostate Cancer Cells: A Prospective Therapeutic Opportunity. Oncotarget.

[132] Korkolopoulou P., Goudopoulou A., Voutsinas G., Thomas-Tsagli E., Kapralos P., Patsouris E., Saetta A.A. (2004). C-FLIP Expression in Bladder Urothelial Carcinomas: Its Role in Resistance to Fas-Mediated Apoptosis and Clinicopathologic Correlations. Urology.

[133] Ullenhag G.J., Mukherjee A., Watson N.F.S., Al-Attar A.H., Scholefield J.H., Durrant L.G. (2007). Overexpression of FLIPL Is an Independent Marker of Poor Prognosis in Colorectal Cancer Patients. Clin. Cancer Res..

[134] Zhang X., Jin T.G., Yang H., Dewolf W.C., Khosravi-Far R., Olumi A.F. (2004). Persistent C-FLIP(L) Expression Is Necessary and Sufficient to Maintain Resistance to Tumor Necrosis Factor-Related Apoptosis-Inducing Ligand-Mediated Apoptosis in Prostate Cancer. Cancer Res..

[135] Bagnoli M., Ambrogi F., Pilotti S., Alberti P., Ditto A., Barbareschi M., Galligioni E., Biganzoli E., Canevari S., Mezzanzanica D. (2009). C-FLIPL Expression Defines Two Ovarian Cancer Patient Subsets and Is a Prognostic Factor of Adverse Outcome. Endocr. Relat. Cancer.

[136] Nam S.Y., Jung G.A., Hur G.C., Chung H.Y., Kim W.H., Seol D.W., Lee B.L. (2003). Upregulation of FLIPs by Akt, a Possible Inhibition Mechanism of TRAIL-Induced Apoptosis in Human Gastric Cancers. Cancer Sci..

[137] El-Gazzar A., Wittinger M., Perco P., Anees M., Horvat R., Mikulits W., Grunt T.W., Mayer B., Krainer M. (2010). The Role of C-FLIPL in Ovarian Cancer: Chaperoning Tumor Cells from Immunosurveillance and Increasing Their Invasive Potential. Gynecol. Oncol..

[138] Zhou X.D., Yu J.P., Liu J., Luo H.S., Chen H.X., Yu H.G. (2004). Overexpression of Cellular FLICE-Inhibitory Protein (FLIP) in Gastric Adenocarcinoma. Clin. Sci..

[139] Chawla-Sarkar M., Bae S.I., Reu F.J., Jacobs B.S., Lindner D.J., Borden E.C. (2004). Downregulation of Bcl-2, FLIP or IAPs (XIAP and Survivin) by SiRNAs Sensitizes Resistant Melanoma Cells to Apo2L/TRAIL-Induced Apoptosis. Cell Death Differ..

[140] Sharp D.A., Lawrence D.A., Ashkenazi A. (2005). Selective Knockdown of the Long Variant of Cellular FLICE Inhibitory Protein Augments Death Receptor-Mediated Caspase-8 Activation and Apoptosis. J. Biol. Chem..

[141] Clarke P., Tyler K.L. (2007). Down-Regulation of CFLIP Following Reovirus Infection Sensitizes Human Ovarian Cancer Cells to TRAIL-Induced Apoptosis P. Apoptosis.

[142] Kim D.Y., Kim M.J., Kim H.B., Lee J.W., Bae J.H., Kim D.W., Kang C.D., Kim S.H. (2011). Suppression of Multidrug Resistance by Treatment with TRAIL in Human Ovarian and Breast Cancer Cells with High Level of C-Myc. Biochim. Biophys. Acta Mol. Basis Dis..

[143] Day T.W., Najafi F., Wu C.H., Safa A.R. (2006). Cellular FLICE-like Inhibitory Protein (c-FLIP): A Novel Target for Taxol-Induced Apoptosis. Biochem. Pharmacol..

[144] Kang J., Bu J., Hao Y., Chen F. (2005). Subtoxic Concentration of Doxorubicin Enhances TRAIL-Induced Apoptosis in Human Prostate Cancer Cell Line LNCaP. Prostate Cancer Prostatic Dis..

[145] Yang B.F., Xiao C., Li H., Yang S.J. (2007). Resistance to Fas-Mediated Apoptosis in Malignant Tumours Is Rescued by KN-93 and Cisplatin via Downregulation of c-FLIP Expression and Phosphorylation. Clin. Exp. Pharmacol. Physiol..

[146] Damia G., Broggini M. (2019). Platinum Resistance in Ovarian Cancer: Role of DNA Repair. Cancers.

[147] Mirza-Aghazadeh-Attari M., Ostadian C., Saei A.A., Mihanfar A., Darband S.G., Sadighparvar S., Kaviani M., Samadi Kafil H., Yousefi B., Majidinia M. (2019). DNA Damage Response and Repair in Ovarian Cancer: Potential Targets for Therapeutic Strategies. DNA Repair.

[148] Hegan D.C., Lu Y., Stacheleka G.C., Crosbya M.E., Bindraa R.S., Glazer P.M. (2010). Inhibition of Poly(ADP-Ribose) Polymerase down-Regulates BRCA1 and RAD51 in a Pathway Mediated by E2F4 and P130. Proc. Natl. Acad. Sci. USA.

[149] D’Andrea A.D. (2018). Mechanisms of PARP Inhibitor Sensitivity and Resistance. DNA Repair.

[150] Ledermann J.A., Drew Y., Kristeleit R.S. (2016). Homologous Recombination Deficiency and Ovarian Cancer. Eur. J. Cancer.

[151] Pascal J.M. (2018). The Comings and Goings of PARP-1 in Response to DNA Damage. DNA Repair.

[152] Swisher E.M., Sakai W., Karlan B.Y., Wurz K., Urban N., Taniguchi T. (2008). Secondary BRCA1 Mutations in BRCA1-Mutated Ovarian Carcinomas with Platinum Resistance. Cancer Res..

[153] Li H., Liu Z.-Y., Wu N., Chen Y.-C., Cheng Q., Wang J. (2020). PARP Inhibitor Resistance: The Underlying Mechanisms and Clinical Implications. Mol. Cancer.

[154] Zhang Z., Dou X., Yang H., Jia L., Qin K., Gao X., Yang B., Zhang W., Qin C., Zhang F. (2020). Association of Expression of P53, Livin, ERCC1, BRCA1 and PARP1 in Epithelial Ovarian Cancer Tissue with Drug Resistance and Prognosis. Pathol. Res. Pract..

[155] Liu Y., Burness M.L., Martin-Trevino R., Guy J., Bai S., Harouaka R., Brooks M.D., Shang L., Fox A., Luther T.K. (2017). RAD51 Mediates Resistance of Cancer Stem Cells to PARP Inhibition in Triple-Negative Breast Cancer. Clin. Cancer Res..

[156] Xiao M., Cai J., Cai L., Jia J., Xie L., Zhu Y., Huang B., Jin D., Wang Z. (2017). Let-7e Sensitizes Epithelial Ovarian Cancer to Cisplatin through Repressing DNA Double Strand Break Repair. J. Ovarian Res..

[157] Patel A.G., Sarkaria J.N., Kaufmann S.H. (2011). Nonhomologous End Joining Drives Poly(ADP-Ribose) Polymerase (PARP) Inhibitor Lethality in Homologous Recombination-Deficient Cells. Proc. Natl. Acad. Sci. USA.

[158] Liu Y.-B., Mei Y., Tian Z.-W., Long J., Luo C.-H., Zhou H.-H. (2018). Downregulation of RIF1 Enhances Sensitivity to Platinum-Based Chemotherapy in Epithelial Ovarian Cancer (EOC) by Regulating Nucleotide Excision Repair (NER) Pathway. Cell. Physiol. Biochem..

[159] Gee M.E., Faraahi Z., McCormick A., Edmondson R.J. (2018). DNA Damage Repair in Ovarian Cancer: Unlocking the Heterogeneity. J. Ovarian Res..

[160] Mei Y., Peng C., Liu Y.B., Wang J., Zhou H.H. (2017). Silencing RIF1 Decreases Cell Growth, Migration and Increases Cisplatin Sensitivity of Human Cervical Cancer Cells. Oncotarget.

[161] Mattarocci S., Hafner L., Lezaja A., Shyian M., Shore D. (2016). Rif1: A Conserved Regulator of DNA Replication and Repair Hijacked by Telomeres in Yeasts. Front. Genet..

[162] Wise H.C., Iyer G.V., Moore K., Temkin S.M., Gordon S., Aghajanian C., Grisham R.N. (2019). Activity of M3814, an Oral DNA-PK Inhibitor, In Combination with Topoisomerase II Inhibitors in Ovarian Cancer Models. Sci. Rep..

[163] Mohiuddin I.S., Kang M.H. (2019). DNA-PK as an Emerging Therapeutic Target in Cancer. Front. Oncol..

[164] Abdel-Fatah T.M.A., Arora A., Moseley P., Coveney C., Perry C., Johnson K., Kent C., Ball G., Chan S., Madhusudan S. (2014). ATM, ATR and DNA-PKcs Expressions Correlate to Adverse Clinical Outcomes in Epithelial Ovarian Cancers. BBA Clin..

[165] Damia G. (2020). Targeting DNA-PK in Cancer. Mutat. Res. Fundam. Mol. Mech. Mutagen..

[166] Dejmek J., Dirk Iglehart J., Lazaro J.B. (2009). DNA-Dependent Protein Kinase (DNA-PK)-Dependent Cisplatin-Induced Loss of Nucleolar Facilitator of Chromatin Transcription (FACT) and Regulation of Cisplatin Sensitivity by DNA-PK and FACT. Mol. Cancer Res..

[167] Saldivar J.S., Wu X., Follen M., Gershenson D. Nucleotide Excision Repair Pathway Review I: Implications in Ovarian Cancer and Platinum Sensitivity. Proceedings of the Gynecologic Oncology.

[168] Li Q., Yu J.J., Mu C., Yunmbam M.K., Slavsky D., Cross C.L., Bostick-Bruton F. (2000). Association between the Level of ERCC-1 Expression and the Repair of Cisplatin-Induced DNA Damage in Human Ovarian Cancer Cells. Anticancer Res..

[169] Arora S., Kothandapani A., Tillison K., Kalman-Maltese V., Patrick S.M. (2010). Downregulation of XPF-ERCC1 Enhances Cisplatin Efficacy in Cancer Cells. DNA Repair.

[170] Mesquita K.A., Alabdullah M., Griffin M., Toss M.S., Fatah T.M.A.A., Alblihy A., Moseley P., Chan S.Y.T., Rakha E.A., Madhusudan S. (2019). ERCC1-XPF Deficiency Is a Predictor of Olaparib Induced Synthetic Lethality and Platinum Sensitivity in Epithelial Ovarian Cancers. Gynecol. Oncol..

[171] Pulzová L.B., Ward T.A., Chovanec M. (2020). XPA: DNA Repair Protein of Significant Clinical Importance. Int. J. Mol. Sci..

[172] Tsodikov O.V., Ivanov D., Orelli B., Staresincic L., Shoshani I., Oberman R., Schärer O.D., Wagner G., Ellenberger T. (2007). Structural Basis for the Recruitment of ERCC1-XPF to Nucleotide Excision Repair Complexes by XPA. EMBO J..

[173] Yu J. (1996). Platinum-Sensitive and Platinum-Resistant Ovarian Cancer Tissues Show Differences in the Relationships between MRNA Levels of P53, ERCC1 and XPA. Int. J. Oncol..

[174] Amable L. (2016). Cisplatin Resistance and Opportunities for Precision Medicine. Pharmacol. Res..

[175] Dabholkar M., Thornton K., Vionnet J., Bostick-Bruton F., Yu J.J., Reed E. (2000). Increased MRNA Levels of Xeroderma Pigmentosum Complementation Group B (XPB) and Cockayne’s Syndrome Complementation Group B (CSB) without Increased MRNA Levels of Multidrug-Resistance Gene (MDR1) or Metallothionein-II (MT-II) in Platinum-Resistant Human. Biochem. Pharmacol..

[176] Rosenberg E., Taher M.M., Kuemmerle N.B., Farnsworth J., Valerie K. (2001). A Truncated Human Xeroderma Pigmentosum Complementation Group A Protein Expressed from an Adenovirus Sensitizes Human Tumor Cells to Ultraviolet Light and Cisplatin Cancer Research. Cancer Res..

[177] Faridounnia M., Folkers G.E., Boelens R. (2018). Function and Interactions of ERCC1-XPF in DNA Damage Response. Molecules.

[178] Kirschner K., Melton D.W. (2010). Multiple Roles of the ERCC1-XPF Endonuclease in DNA Repair and Resistance to Anticancer Drugs. Anticancer Res..

[179] Pettitt S.J., Krastev D.B., Brandsma I., Dréan A., Song F., Aleksandrov R., Harrell M.I., Menon M., Brough R., Campbell J. (2018). Genome-Wide and High-Density CRISPR-Cas9 Screens Identify Point Mutations in PARP1 Causing PARP Inhibitor Resistance. Nat. Commun..

[180] Abdel-Fatah T., Sultana R., Abbotts R., Hawkes C., Seedhouse C., Chan S., Madhusudan S. (2013). Clinicopathological and Functional Significance of XRCC1 Expression in Ovarian Cancer. Int. J. Cancer.

[181] Zhang Z., Xie Z., Sun G., Yang P., Li J., Yang H., Xiao S., Liu Y., Qiu H., Qin L. (2015). Reversing Drug Resistance of Cisplatin by Hsp90 Inhibitors in Human Ovarian Cancer Cells. Int. J. Clin. Exp. Med..

[182] Boudsocq F., Benaim P., Canitrot Y., Knibiehler M., Ausseil F., Capp J.P., Bieth A., Long C., David B., Shevelev I. (2005). Modulation of Cellular Response to Cisplatin by a Novel Inhibitor of DNA Polymerase β. Mol. Pharmacol..

[183] Bergoglio V., Canitrot Y., Hogarth L., Minto L., Howell S.B., Cazaux C., Hoffmann J.S. (2001). Enhanced Expression and Activity of DNA Polymerase β in Human Ovarian Tumor Cells: Impact on Sensitivity towards Antitumor Agents. Oncogene.

[184] Nemec A.A., Abriola L., Merkel J.S., De Stanchina E., DeVeaux M., Zelterman D., Glazer P.M., Sweasy J.B. (2017). DNA Polymerase Beta Germline Variant Confers Cellular Response to Cisplatin Therapy. Mol. Cancer Res..

[185] Sawant A., Floyd A.M., Dangeti M., Lei W., Sobol R.W., Patrick S.M. (2017). Differential Role of Base Excision Repair Proteins in Mediating Cisplatin Cytotoxicity. DNA Repair.

[186] Chiruvella K.K., Liang Z., Wilson T.E. (2013). Repair of Double-Strand Breaks by End Joining. Cold Spring Harb. Perspect. Biol..

[187] Kondrashova O., Topp M., Nesic K., Lieschke E., Ho G.Y., Harrell M.I., Zapparoli G.V., Hadley A., Holian R., Boehm E. (2018). Methylation of All BRCA1 Copies Predicts Response to the PARP Inhibitor Rucaparib in Ovarian Carcinoma. Nat. Commun..

[188] Labidi-Galy S.I., Olivier T., Rodrigues M., Ferraioli D., Derbel O., Bodmer A., Petignat P., Rak B., Chopin N., Tredan O. (2018). Location of Mutation in BRCA2 Gene and Survival in Patients with Ovarian Cancer. Clin. Cancer Res..

[189] Rivera B., Di Iorio M., Frankum J., Nadaf J., Fahiminiya S., Arcand S.L., Burk D.L., Grapton D., Tomiak E., Hastings V. (2017). Functionally Null RAD51D Missense Mutation Associates Strongly with Ovarian Carcinoma. Cancer Res..

[190] Kondrashova O., Nguyen M., Shield-Artin K., Tinker A.V., Teng N.N.H., Harrell M.I., Kuiper M.J., Ho G.Y., Barker H., Jasin M. (2017). Secondary Somatic Mutations Restoring RAD51C and RAD51D Associated with Acquired Resistance to the PARP Inhibitor Rucaparib in High-Grade Ovarian Carcinoma. Cancer Discov..

[191] Ahmed N., Abubaker K., Findlay J.K. (2014). Ovarian Cancer Stem Cells: Molecular Concepts and Relevance as Therapeutic Targets. Mol. Aspects Med..

[192] Mihanfar A., Aghazadeh Attari J., Mohebbi I., Majidinia M., Kaviani M., Yousefi M., Yousefi B. (2019). Ovarian Cancer Stem Cell: A Potential Therapeutic Target for Overcoming Multidrug Resistance. J. Cell. Physiol..

[193] Burgos-Ojeda D., Rueda B.R., Buckanovich R.J. (2012). Ovarian Cancer Stem Cell Markers: Prognostic and Therapeutic Implications. Cancer Lett..

[194] Motohara T., Katabuchi H. (2019). Ovarian Cancer Stemness: Biological and Clinical Implications for Metastasis and Chemotherapy Resistance. Cancers.

[195] Piva M., Domenici G., Iriondo O., Rábano M., Simões B.M., Comaills V., Barredo I., López-Ruiz J.A., Zabalza I., Kypta R. (2014). Sox2 Promotes Tamoxifen Resistance in Breast Cancer Cells. EMBO Mol. Med..

[196] Chen B., Zhu Z., Li L., Ye W., Zeng J., Gao J., Wang S., Zhang L., Huang Z. (2019). Effect of Overexpression of Oct4 and Sox2 Genes on the Biological and Oncological Characteristics of Gastric Cancer Cells. Onco. Targets. Ther..

[197] Wang X., Ji X., Chen J., Yan D., Zhang Z., Wang Q., Xi X., Feng Y. (2014). SOX2 Enhances the Migration and Invasion of Ovarian Cancer Cells via Src Kinase. PLoS ONE.

[198] Belotte J., Fletcher N.M., Alexis M., Morris R.T., Munkarah A.R., Diamond M.P., Saed G.M. (2015). Sox2 Gene Amplification Significantly Impacts Overall Survival in Serous Epithelial Ovarian Cancer. Reprod. Sci..

[199] Ye F., Li Y., Hu Y., Zhou C., Hu Y., Chen H. (2011). Expression of Sox2 in Human Ovarian Epithelial Carcinoma. J. Cancer Res. Clin. Oncol..

[200] Zhang J., Chang D.Y., Mercado-Uribe I., Liu J. (2012). Sex-Determining Region Y-Box 2 Expression Predicts Poor Prognosis in Human Ovarian Carcinoma. Hum. Pathol..

[201] Bareiss P.M., Paczulla A., Wang H., Schairer R., Wiehr S., Kohlhofer U., Rothfuss O.C., Fischer A., Perner S., Staebler A. (2013). SOX2 Expression Associates with Stem Cell State in Human Ovarian Carcinoma. Cancer Res..

[202] Wen Y., Hou Y., Huang Z., Cai J., Wang Z. (2017). SOX2 Is Required to Maintain Cancer Stem Cells in Ovarian Cancer. Cancer Sci..

[203] Cox J.L., Mallanna S.K., Luo X., Rizzino A. (2010). Sox2 Uses Multiple Domains to Associate with Proteins Present in Sox2-Protein Complexes. PLoS ONE.

[204] Wang J., Rao S., Chu J., Shen X., Levasseur D.N., Theunissen T.W., Orkin S.H. (2006). A Protein Interaction Network for Pluripotency of Embryonic Stem Cells. Nature.

[205] Ben-Porath I., Thomson M.W., Carey V.J., Ge R., Bell G.W., Regev A., Weinberg R.A. (2008). An Embryonic Stem Cell–like Gene Expression Signature in Poorly Differentiated Aggressive Human Tumors. Nat. Genet..

[206] Nagata T., Shimada Y., Sekine S., Hori R., Matsui K., Okumura T., Sawada S., Fukuoka J., Tsukada K. (2014). Prognostic Significance of NANOG and KLF4 for Breast Cancer. Breast Cancer.

[207] Liu S., Sun J., Cai B., Xi X., Yang L., Zhang Z., Feng Y., Sun Y. (2016). NANOG Regulates Epithelial-Mesenchymal Transition and Chemoresistance through Activation of the STAT3 Pathway in Epithelial Ovarian Cancer. Tumor Biol..

[208] Peng S., Maihle N.J., Huang Y. (2010). Pluripotency Factors Lin28 and Oct4 Identify a Sub-Population of Stem Cell-like Cells in Ovarian Cancer. Oncogene.

[209] Meng H.M., Zheng P., Wang X.Y., Liu C., Sui H.M., Wu S.J., Zhou J., Ding Y.Q., Li J.M. (2010). Overexpression of Nanog Predicts Tumor Progression and Poor Prognosis in Colorectal Cancer. Cancer Biol. Ther..

[210] Zhang S., Balch C., Chan M.W., Lai H.-C., Matei D., Schilder J.M., Yan P.S., Huang T.H., Nephew K.P. (2008). Identification and Characterization of Ovarian Cancer-Initiating Cells from Primary Human Tumors. Cancer Res..

[211] Lee M., Nam E.J., Kim S.W., Kim S., Kim J.H., Kim Y.T. (2012). Prognostic Impact of the Cancer Stem Cell-Related Marker NANOG in Ovarian Serous Carcinoma. Int. J. Gynecol. Cancer.

[212] Lu Y., Zhu H., Shan H., Lu J., Chang X., Li X., Lu J., Fan X., Zhu S., Wang Y. (2013). Knockdown of Oct4 and Nanog Expression Inhibits the Stemness of Pancreatic Cancer Cells. Cancer Lett..

[213] Abubaker K., Luwor R.B., Zhu H., McNally O., Quinn M.A., Burns C.J., Thompson E.W., Findlay J.K., Ahmed N. (2014). Inhibition of the JAK2/STAT3 Pathway in Ovarian Cancer Results in the Loss of Cancer Stem Cell-like Characteristics and a Reduced Tumor Burden. BMC Cancer.

[214] Abubaker K., Luwor R.B., Escalona R., McNally O., Quinn M.A., Thompson E.W., Findlay J.K., Ahmed N. (2014). Targeted Disruption of the JAK2/STAT3 Pathway in Combination with Systemic Administration of Paclitaxel Inhibits the Priming of Ovarian Cancer Stem Cells Leading to a Reduced Tumor Burden. Front. Oncol..

[215] Quintás-Cardama A., Verstovsek S. (2013). Molecular Pathways: JAK/STAT Pathway: Mutations, Inhibitors, and Resistance. Clin. Cancer Res..

[216] Reeves P.M., Abbaslou M.A., Kools F.R.W., Vutipongsatorn K., Tong X., Gavegnano C., Schinazi R.F., Poznansky M.C. (2018). Correction: Ruxolitinib Sensitizes Ovarian Cancer to Reduced Dose Taxol, Limits Tumor Growth and Improves Survival in Immune Competent Mice. Oncotarget.

[217] Han E.S., Wen W., Dellinger T.H., Wu J., Lu S.A., Jove R., Yim J.H. (2018). Ruxolitinib Synergistically Enhances the Anti-Tumor Activity of Paclitaxel in Human Ovarian Cancer. Oncotarget.

[218] Áyen Á., Martínez Y.J., Marchal J.A., Boulaiz H. (2018). Recent Progress in Gene Therapy for Ovarian Cancer. Int. J. Mol. Sci..

[219] Stein M.N., Malhotra J., Tarapore R.S., Malhotra U., Silk A.W., Chan N., Rodriguez L., Aisner J., Aiken R.D., Mayer T. (2019). Safety and Enhanced Immunostimulatory Activity of the DRD2 Antagonist ONC201 in Advanced Solid Tumor Patients with Weekly Oral Administration. J. Immunother. Cancer.

[220] Stein M.N., Bertino J.R., Kaufman H.L., Mayer T., Moss R., Silk A., Chan N., Malhotra J., Rodriguez L., Aisner J. (2017). First-in-Human Clinical Trial of Oral ONC201 in Patients with Refractory Solid Tumors. Clin. Cancer Res..

[221] Brenner A.J., Cohen Y.C., Breitbart E., Bangio L., Sarantopoulos J., Giles F.J., Borden E.C., Harats D., Triozzi P.L. (2013). Phase i Dose-Escalation Study of VB-111, an Antiangiogenic Virotherapy, in Patients with Advanced Solid Tumors. Clin. Cancer Res..

[222] Blagden S.P., Hamilton A.L., Mileshkin L., Wong S., Michael A., Hall M., Goh J.C., Lisyanskaya A.S., DeSilvio M., Frangou E. (2019). Phase IB Dose Escalation and Expansion Study of Akt Inhibitor Afuresertib with Carboplatin and Paclitaxel in Recurrent Platinum-Resistant Ovarian Cancer. Clin. Cancer Res..

[223] Risnayanti C., Jang Y.S., Lee J., Ahn H.J. (2018). PLGA Nanoparticles Co-Delivering MDR1 and BCL2 SiRNA for Overcoming Resistance of Paclitaxel and Cisplatin in Recurrent or Advanced Ovarian Cancer. Sci. Rep..

[224] Vaghari-Tabari M., Hassanpour P., Sadeghsoltani F., Malakoti F., Alemi F., Qujeq D., Asemi Z., Yousefi B. (2022). CRISPR/Cas9 Gene Editing: A New Approach for Overcoming Drug Resistance in Cancer. Cell. Mol. Biol. Lett..

[225] Norouzi-Barough L., Sarookhani M., Salehi R., Sharifi M., Moghbelinejad S. (2018). CRISPR/Cas9, a New Approach to Successful Knockdown of ABCB1/P-Glycoprotein and Reversal of Chemosensitivity in Human Epithelial Ovarian Cancer Cell Line. Iran. J. Basic Med. Sci..

[226] Sterner R.C., Sterner R.M. (2021). CAR-T Cell Therapy: Current Limitations and Potential Strategies. Blood Cancer J..

[227] Zhu X., Cai H., Zhao L., Ning L., Lang J. (2017). CAR-T Cell Therapy in Ovarian Cancer: From the Bench to the Bedside. Oncotarget.

[228] Le Saux O., Ray-Coquard I., Labidi-Galy S.I. (2021). Challenges for Immunotherapy for the Treatment of Platinum Resistant Ovarian Cancer. Semin. Cancer Biol..

[229] Marofi F., Motavalli R., Safonov V.A., Thangavelu L., Yumashev A.V., Alexander M., Shomali N., Chartrand M.S., Pathak Y., Jarahian M. (2021). CAR T Cells in Solid Tumors: Challenges and Opportunities. Stem Cell Res. Ther..

